# The protein kinase complex CBL10–CIPK8–SOS1 functions in Arabidopsis to regulate salt tolerance

**DOI:** 10.1093/jxb/erz549

**Published:** 2019-12-10

**Authors:** Xiaochang Yin, Youquan Xia, Qing Xie, Yuxin Cao, Zhenyu Wang, Gangping Hao, Jie Song, Yang Zhou, Xingyu Jiang

**Affiliations:** 1 Hainan Key Laboratory for Sustainable Utilization of Tropical Bioresources/Hainan Key Laboratory for Biotechnology of Salt Tolerant Crops/Institute of Tropical Agriculture and Forestry, Hainan University, Haikou, China; 2 School of Life Science, Taishan Medical University, Tai’an, China; 3 Shandong Key Laboratory of Plant Stress/College of Life Science, Shandong Normal University, Jinan, China; 4 University of Maryland, USA

**Keywords:** Arabidopsis, calcineurin B-like protein 10, CBL-interacting protein kinase 8, salt stress, salt tolerance, SOS pathway

## Abstract

Salt tolerance in plants is mediated by Na^+^ extrusion from the cytosol by the plasma membrane Na^+^/H^+^ antiporter SOS1. This is activated in Arabidopsis root by the protein kinase complex SOS2–SOS3 and in Arabidopsis shoot by the protein kinase complex CBL10–SOS2, with SOS2 as a key node in the two pathways. The *sos1* mutant is more sensitive than the *sos2* mutant, suggesting that other partners may positively regulate SOS1 activity. Arabidopsis has 26 CIPK family proteins of which CIPK8 is the closest homolog to SOS2. It is hypothesized that CIPK8 can activate Na^+^ extrusion by SOS1 similarly to SOS2. The plasma membrane Na^+^/H^+^ exchange activity of transgenic yeast co-expressing *CBL10*, *CIPK8*, and *SOS1* was higher than that of untransformed and *SOS1* transgenic yeast, resulting in a lower Na^+^ accumulation and a better growth phenotype under salinity. However, CIPK8 could not interact with SOS3, and the co-expression of *SOS3*, *CIPK8*, and *SOS1* in yeast did not confer a significant salt tolerance phenotype relative to *SOS1* transgenic yeast. Interestingly, *cipk8* displayed a slower Na^+^ efflux, a higher Na^+^ level, and a more sensitive phenotype than wild-type Arabidopsis, but grew better than *sos2* under salinity stress. As expected, *sos2cipk8* exhibited a more severe salt damage phenotype relative to *cipk8* or *sos2*. Overexpression of *CIPK8* in both *cipk8* and *sos2cipk8* attenuated the salt sensitivity phenotype. These results suggest that CIPK8-mediated activation of SOS1 is CBL10-dependent and SOS3-independent, indicating that CIPK8 and SOS2 activity in shoots is sufficient for regulating Arabidopsis salt tolerance.

## Teaser

In Arabidopsis, the Na^+^/H^+^ antiporter SOS1, which functions in salt tolerance, is activated by the protein kinase complex CBL10–CIPK8 in shoots, independent of the root activator SOS3.

## Introduction

Conditions of high soil salinity are highly unfavorable for agricultural productivity because high salt concentrations in the soil are detrimental to plant growth and development. Na^+^ is the predominant ion in the vast majority of saline soil areas. Plants will readily take up Na^+^ from the soil, and excessive accumulation of cytosolic Na^+^ is toxic to several important metabolic processes (Ji *et al.*, 2013; Song and Wang, 2015). Therefore, it is essential for plants to have a way to prevent or decrease cytosolic Na^+^ accumulation in response to salt injury (Clarkson and Hanson, 1980; Tester and Davenport, 2003; Song *et al.*, 2011; Duan *et al.*, 2018). There are several known mechanisms by which plants are able to protect themselves from excessive accumulation of Na^+^ in the cytoplasm. Plants can maintain the cytoplasmic Na^+^ at a low concentration by restricting Na^+^ entry into cells, although the specific mechanisms that inhibit Na^+^ entry are still unclear. Alternatively, Na^+^ can be compartmentalized in the vacuole or exported by plasma membrane transporters. Na^+^ extrusion mediated by the plasma membrane Na^+^/H^+^ antiporter salt overly sensitive 1 (SOS1) is the most efficient at maintaining Na^+^ at a non-toxic level in the cytoplasm (Ji *et al.*, 2013). Of the known plant SOS1 proteins, Arabidopsis SOS1 was the first plasma membrane Na^+^/H^+^ antiporter to be thoroughly characterized physiologically, biochemically, and molecularly (Wu *et al.*, 1996; Quintero *et al.*, 2011).

*SOS1* is highly conserved across the plant kingdom. Salinity stress upregulates its expression in rice (*Oryza sativa*) and Arabidopsis, and its transcript levels increase in wheat plants grown in high salt environments (Shi *et al.*, 2000; Martínez-Atienza *et al.*, 2007; Xu *et al.*, 2008). *Thellungiella salsuginea* (a halophytic relative of Arabidopsis) has higher *SOS1* mRNA levels than Arabidopsis upon exposure to salinity stress (Oh *et al.*, 2009). *SOS1*-knockout Arabidopsis plants are highly sensitive to salt treatment (Wu *et al.*, 1996; Liu and Zhu, 1997; Zhu *et al.*, 1998). The growth of *SOS1*-RNAi (RNA interference) *T. salsuginea* plants is inhibited by salt (Oh *et al.*, 2009). The expression of endogenous *SOS1* and of homologous *SOS1* from other plant species can rescue the salt sensitivity phenotype of the Arabidopsis *sos1* mutant (Shi *et al.*, 2000; Martínez-Atienza *et al.*, 2007). Arabidopsis overexpressing *SOS1* demonstrates better growth ability compared with wild-type plants when treated with NaCl (Shi *et al.*, 2003). Expression of wheat *SOS1* improves the growth of transgenic tobacco (*Nicotiana tabacum*) exposed to NaCl treatment (Zhou *et al.*, 2016). These findings indicated that SOS1 is a key player in mediating plant halotolerance.

In the absence of salt stress, SOS1 is maintained in an inactive state because the C-terminal auto-inhibitory domain interacts with and occupies the adjacent activation domain (Quintero *et al.*, 2011; Zhou *et al.*, 2016). Under conditions of salinity stress, a calcium sensor protein, SOS3 (also known as Calcineurin B-like 4, CBL4), binds Ca^2+^ and subsequently complexes with and activates the protein kinase SOS2 (also known as CBL-interacting protein kinase 24, CIPK24). This Ca^2+^-dependent SOS2–SOS3 protein kinase complex then phosphorylates SOS1 to relieve its autoinhibition. The activated SOS1 transports Na^+^ out of cells using the energy from the proton gradient generated by the H^+^-ATPase (Shi *et al.*, 2000; Qiu *et al.*, 2002; Quintero *et al.*, 2002, 2011; Zhou *et al.*, 2015). Likewise in the response of rice to salinity stress, CBL4 interacts with CIPK24, resulting in the phosphorylation and activation of Na^+^/H^+^ exchange through SOS1 (Martínez-Atienza *et al.*, 2007). The SOS pathway is highly conserved in woody plants; for example in poplar (*Populus trichocarpa*), SOS2–SOS3 interaction recruits SOS2 to the plasma membrane, leading to activation of the downstream target SOS1 (Tang *et al.*, 2010).

While in Arabidopsis roots exposed to increased salinity, the activated protein kinase complex composed of SOS3 and SOS2 activates SOS1, in shoots CBL10 (also known as SOS3-like calcium binding protein 8, SCABP8) interacts with and phosphorylates SOS2, and the phosphorylated SOS2 complex then promotes Na^+^ extrusion mediated by SOS1 (Quan *et al.*, 2007).

Therefore, the SOS3/CBL10–SOS2–SOS1 signaling pathways are the paramount regulatory mechanisms for facilitating Na^+^ extrusion and are critical to the ability of plants to adapt to and tolerate conditions of increased soil salinity. Mutations in *CBL10*, *SOS3*, *SOS2*, or *SOS1* all result in Arabidopsis plants that are sensitive to increased salinity, with *sos1* exhibiting the most severe growth defect among the four *sos* pathway mutants (Wu *et al.*, 1996; Zhu *et al.*, 1998; Quan *et al.*, 2007). This finding opens the possibility that novel components may be involved in activating SOS1 to promote salt tolerance in plants. Interestingly, *cbl10cipk24* double mutants were more sensitive to salt stress than *cipk24*, suggesting that CBL10 may coordinate additional components besides CIPK24 (SOS2) to promote salt tolerance, but a salt-tolerance pathway regulated by CBL10 has yet to be discovered (Yang *et al.*, 2019). There are 26 CIPK family members in Arabidopsis; of these, CIPK8 is the closest protein kinase homolog to CIPK24 (SOS2), so we hypothesized that CIPK8 may be an alternative regulator of SOS1 activity. To examine this hypothesis, we explored how CIPK8 functions in the context of high salinity and tested the interactions among CBLs, CIPK8 and SOS1. We employed yeast complementation and two-hybrid techniques to test our hypothesis and further verify the regulation of SOS1 activity by CBLs–CIPK8 networks. Genetic analysis combined with biochemical and phenotypic studies of the *cipk8* mutant demonstrated that the CBL10–CIPK8 complex activates Na^+^ extrusion of SOS1 in Arabidopsis shoots, thereby promoting the salt tolerance capability of Arabidopsis.

## Materials and methods

### Plant materials and salt treatment

A *cipk8* T-DNA insertion line (SALK_139697) was obtained from the Arabidopsis Biological Resource Center (ABRC) and confirmed using the gene-specific primers LP, RP and the T-DNA left border primer LBb1.3. Both the *sos1* and *sos2* mutants were of the Col-0 background, as described in a previous report (Martínez-Atienza *et al.*, 2007). The double mutant *sos2cipk8* was obtained by crossing *cipk8* to *sos2*. DNA was extracted from wild-type and mutant plants using TRIzol (Thermo Fisher Scientific, Waltham, MA, USA). Polymerase chain reaction (PCR) with gene-specific primers and template genomic DNA was used to identify the above mutants (see Supplementary Table S1 at *JXB* online). All PCR products were separated on 1% agarose gels.

Arabidopsis seeds were germinated on MS plates in a growth chamber at 22 °C using a 16 h light–8 h dark cycle. Five-day-old seedlings were then transferred onto MS plates containing different concentrations of NaCl and allowed to grow for 7 d. After 7 d the seedlings were photographed, and their growth parameters (root length and fresh weight) were recorded. To directly evaluate the salinity stress response, Arabidopsis plants were grown in pots with soil in the greenhouse at 22 °C with 12 h light–12 h dark cycles for 3 weeks. The plants were then treated with 150 mM NaCl for 4 weeks, after which they were photographed and weighted.

### Gene expression assay

Total RNA was extracted from various plant tissues (roots, stems, rosette leaves, cauline leaves, flowers and siliques) of Arabidopsis plants using TRIzol reagent (Thermo Fisher Scientific). One microgram of total RNA was subjected to the reverse transcription reaction using the PrimeScript RT reagent kit with gDNA Eraser (TaKaRa, Dalian, China). *CIPK8* expression level in different tissues was analysed by RT-PCR with primers (CIPK8-RT-F and CIPK8-RT-R, Supplementary Table S1) using template cDNA. The housekeeping gene *actin* was used as an internal control.

In order to analyse the effect of salt stress on *CIPK8* expression, Arabidopsis seedlings were treated with 100 mM NaCl. Total RNA was extracted from the seedlings at different time points post-NaCl treatment. Quantitative real-time PCR (qRT-PCR) analysis was performed with the ABI7900HT system using 2×SYBR Premix Ex Taq II reagents (TaKaRa) with the *actin* gene as an internal control, according to the manufacturer’s protocol. The primers for the amplifications of *CIPK8* (CIPK8-qRT-F and CIPK8-qRT-R) and *actin* (Actin-qRT-F and Actin-qRT-R) genes are listed in Supplementary Table S1. PCR conditions were as follows: 95 °C for 30 s, followed by 40 cycles of 95 °C for 5 s, 60 °C for 30 s. A dissociation curve from 60 °C to 95 °C was generated to verify primer specificity. The data were analysed using SDS plate utility software version 2.4.

### Subcellular localization of CIPK8 and SOS2

The full length *CIPK8* and *SOS2* genes lacking the stop codon were made using PCR with the primers CIPK8-GFP-F/R or SOS2-GFP-F/R (see Supplementary Table S1) and cDNA from wild-type plants. These oligos were then fused with *GFP* in the vector pCAMBIA1300. The resulting constructs, pCAMBIA1300-CIPK8-GFP and pCAMBIA1300-SOS2-GFP were separately, transformed into Arabidopsis mesophyll protoplasts. For co-localization experiments, the *CIPK8* and *SOS2* genes lacking the stop codon were made using PCR with the primers CIPK8-221-F/R or SOS2-221-F/R (Supplementary Table S1), and then fused with *YFP* or *CFP* in the vector pENSG via Gateway system cloning. The resulting constructs, pENSG-CIPK8-YFP and pENSG-SOS2-CFP, were co-transformed into both tobacco (*Nicotiana benthamiana*) leaf cells and Arabidopsis mesophyll protoplasts. Green fluorescent protein (GFP), yellow fluorescent protein (YFP), or cyan fluorescent protein (CFP) fluorescence signals in the leaf epidermal cells and protoplasts were detected using a confocal laser scanning microscope (FV3000; Olympus Corp., Tokyo, Japan).

### Assays for the expression of reporter gene *GUS*

The 1500 bp sequence upstream of the translation start site of *CIPK8* was selected as the promoter region (proCIPK8) and amplified from template genomic DNA using the primers CIPK8P-F and CIPK8P-R (see Supplementary Table S1). The resulting DNA fragment was cloned into the *Kpn*I and *Nco*I sites of the pCAMBIA1301 vector containing the *GUS* reporter gene. The recombinant plasmid was introduced into Arabidopsis using *Agrobacterium tumefaciens* strain GV3101. The expression level of the proCIPK8-driven reporter gene was analysed in various tissues from transgenic plants using the β-glucuronidase (GUS) staining method (Jefferson *et al.*, 1987).

### Complementation assay for *cipk8* mutation

To test for complementation of the *cipk8* salt sensitivity phenotype, a 5502 bp genomic DNA fragment containing 2399 bp upstream of the start codon of *CIPK8* was obtained by PCR from template genomic DNA using the primers CIPK8P-CIPK8-F and CIPK8P-CIPK8-R (see Supplementary Table S1). The resulting fragment was inserted into the vector pCAMBIA1300 between *Kpn*I and *Sal*I sites. The recombinant plasmid pCAMBIA1300-proCIPK8 was introduced into *A. tumefaciens* GV3101 by electroporation and transformed into both *cipk8* and *sos2cipk8* by the floral dip method (Clough and Bent, 1998).

### Interactions between CIPK8 and CBL proteins

The full length *CIPK8* and 10 *CBL* genes were amplified from Arabidopsis plant-derived cDNA by PCR with their respective primers (CIPK8-BD-F/R, CBL1-AD-F/R and CBL10-AD-F/R; Supplementary Table S1) and inserted into pGBKT7 and pGADT7, respectively. The pGBKT7-*CIPK8* and pGADT7-*CBL*s vectors were co-transformed into the Y2HGold yeast (*Saccharomyces cerevisiae*) strain to examine their interactions using the MatchMaker yeast two-hybrid system (Clontech, USA).

Interactions in plant cells between CIPK8 and CBL10, CIPK8 and SOS3, and SOS2 and CBL10 were tested using the Split-LUC assay (Jing *et al.*, 2016). The coding sequences of these genes were amplified by PCR from Arabidopsis plant cDNA (primers: CIPK8-cLUC-F/R, SOS2-cLUC-F/R, CBL10-nLUC-F/R, and SOS3-nLUC-F/R; Supplementary Table S1) and cloned into pCAMBIA1300-nLUC or pCAMBIA1300-cLUC to generate N-terminal or C-terminal luciferase-fusion constructs, respectively. The resulting constructs were electroporated into *A. tumefaciens* GV3101. Agrobacterial suspensions were infiltrated into the fully expanded leaves of 7-week-old *Nicotiana benthamiana* plants using a needleless syringe. After 2 d of infiltration, the leaves were incubated in the dark for 10 min, after which luciferase activity was detected using a luminescence imaging system with a 10 min exposure time (Jing *et al.*, 2016).

### Yeast test

The *Saccharomyces cerevisiae* mutant strain AXT3K (*4ena1::HIS3::4ena4*, *4nha1::LEU2*, and *4nhx1::KanMX4*), which lacks the main plasma membrane Na^+^ transporters (Quintero *et al.*, 2011; Zhou *et al.*, 2015), was used to test CIPK8 function. The coding sequences of *SOS1* and *CIPK8* were amplified by PCR using cDNA from Arabidopsis plants (primers: SOS1-p416-F/R and CIPK8-p414-F/R, Supplementary Table S1) and cloned into the yeast expression vectors p416- and p414-GPD, respectively. In order to co-express the *CIPK8* and *CBL* genes or the *SOS2* and *CBL* genes, the bicistronic plasmids p414-*GPD*:*CIPK8(SOS2):CYC1*-*GPD*:*CBLs:CYC1* were constructed using the overlap extension PCR (SOE-PCR) method (Heckman and Pease, 2007). Site-directed mutagenesis of serines 1136 and 1138 in the *SOS1* gene (DS_1136_PS_1138_) to alanine (A) was carried out using PCR with p416-*SOS1* as the template (primers: SOS1-p416-F and 1136A-p416-R for DA_1136_PS_1138_, SOS1-p416-F and 1138A-p416-R for DS_1136_PA_1138_, and SOS1-p416-F and 1136A1138A-p416-R for DA_1136_PA_1138_; Supplementary Table S1). These constructs were introduced into AXT3K using the PEG/LiAc method (Zhou *et al.*, 2015).

Salt tolerance tests were performed in AP medium (8 mM phosphoric acid, 10 mM arginine, 2% glucose, 2 mM MgSO_4_, 1 mM KCl, 0.2 mM CaCl_2_, plus trace elements and vitamins, adjusted to pH 6.5 with arginine). Transgenic and untransformed yeast cells were precultured to saturation in liquid AP medium. The saturated medium was diluted, and 10 µl aliquots of each serial dilution were spotted onto AP plates supplemented with the indicated concentration of NaCl and allowed to grow for 3–5 d at 28 °C.

### Na^+^/H^+^ exchange activity and Na^+^ content determination

Yeast cells transformed with *SOS1* alone, co-transformed with *SOS1*, *CIPK8*, and *CBL10* genes or non-transformed were incubated in 1 liter of AP medium at 28 °C with shaking (200 rpm) until the culture reached saturation. Upon reaching saturation, 100 mM NaCl was added to the cultures, which were then incubated for another 1 h. Yeast cells were then harvested by centrifugation, and the plasma membranes were extracted using an aqueous two-phase system. Plasma membrane Na^+^/H^+^ exchange activity was assayed using a fluorescence spectrophotometer (Hitachi F-2500, Japan) (Martínez-Atienza *et al.*, 2007; Xu *et al.*, 2008).

Yeast cells were grown either with or without 30 mM NaCl in 2.5 l of AP medium at 28 °C with shaking (200 rpm). The cells were harvested by centrifugation (3000 *g*, 5 min) when the OD_600_ reached 0.25±0.01. After treatment with HEPES buffer (Zhou *et al.*, 2016), the cells’ sodium content was determined using an atomic absorption spectrometer (AA-670, Shimadzu Corp., Kyoto, Japan).

### Determination of Na^+^ flux and Na^+^ content of leaves

WT and *cipk8* mutant seeds were germinated on MS plates at 22 °C. Seven-day-old seedlings were transferred to MS medium containing 100 mM NaCl and allowed to grow for 24 h. Then half leaves with hand-cut cross section from the salt-stressed seedlings were equilibrated in buffer for 5 min, after which net Na^+^ flux at a cross section of the leaves was determined in fresh buffer using non-invasive micro-test technology (NMT100 Series, Younger USA LLC, Amherst, MA, USA) and iFluxes/imFluxes 1.0 software (Younger USA) as described previously (Sun *et al.*, 2009; Zhou *et al.*, 2016; Fan *et al.*, 2019) in the Younger USA (Xuyue Beijing) NMT Service Center.

After treatment with 150 mM NaCl in soil for 4 weeks, the leaves from WT and *cipk8* mutant plants were collected. The Na^+^ level in the collected leaf samples was measured using atomic absorption spectrometry as described previously (Zhou *et al.*, 2016).

### Statistical analysis

A two-tailed Student’s *t*-test was used to analyse the data. The results are expressed as the mean ±SE, and differences with a *P*-value<0.05 were considered statistically significant. At least three biological replicates were used for each experiment.

## Results

### Phylogenetic analysis and subcellular localization of CIPK8 protein

The SOS pathways, which comprise four components, SOS3/CBL10, SOS2 and SOS1, are essential for protecting Arabidopsis from salt stress. Under conditions of increased salinity, SOS3 or CBL10 interacts with the kinase SOS2 and promotes the Na^+^/H^+^ exchange activity of SOS1. Loss-of-function mutants for these SOS components are all sensitive to salt stress (Wu *et al.*, 1996; Martínez-Atienza *et al.*, 2007; Quan *et al.*, 2007), and comparative analysis revealed the *sos1* mutant is more sensitive than the *sos2* mutant (see Supplementary Fig. S1), indicating that SOS2 alone is not enough to completely activate SOS1 activity. This finding is highly suggestive that there may be other components that positively regulate the function of SOS1. Bioinformatics analysis of the Arabidopsis genome has unraveled complex signaling networks comprising 10 CBL-type calcium sensors and 26 CIPK-type target kinases in Arabidopsis (Kolukisaoglu *et al.*, 2004; Weinl and Kudla, 2009). Among the 26 CIPK proteins, CIPK8 is the closest homolog to SOS2 (CIPK24) (Supplementary Fig. S2).

**Fig. 1.**
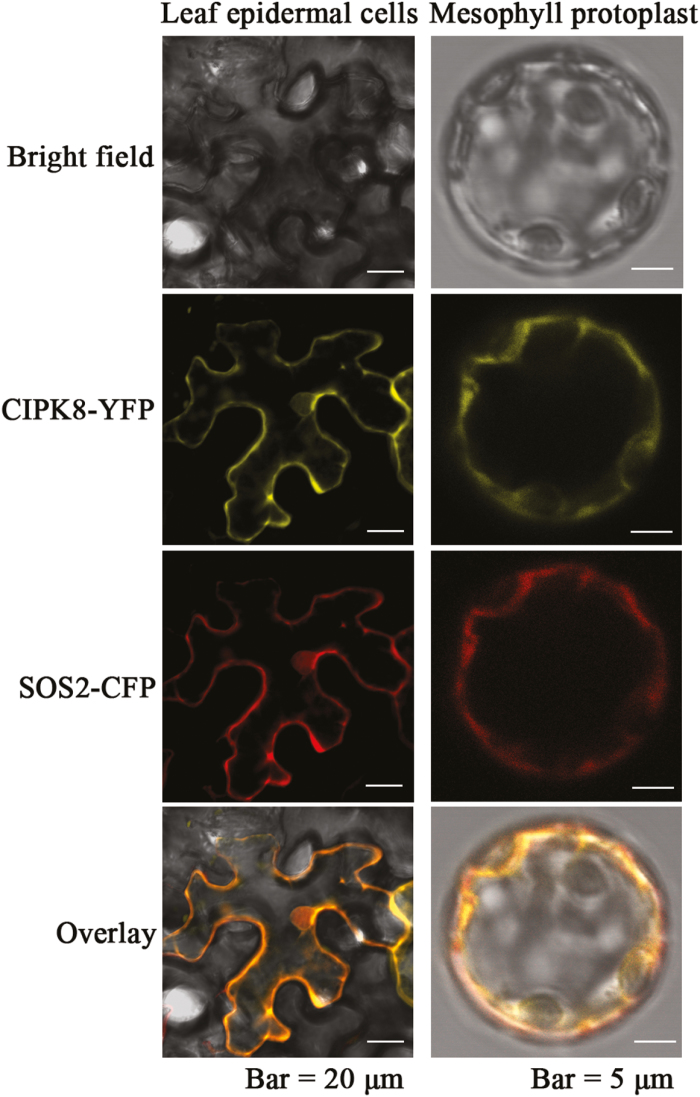
Co-localization of CIPK8 and SOS2 in tobacco leaf epidermal cells and Arabidopsis protoplasts. Plasmids expressing the CIPK8–YFP and SOS2–CFP fusion proteins were produced as described in ‘Materials and methods’. The plasmids were transformed into tobacco leaf cells and Arabidopsis protoplasts. The fluorescence signals from tobacco epidermal cells (left panel; scale bars: 20 μm) and Arabidopsis mesophyll protoplasts (right panel; scale bars: 5 μm) were detected using confocal microscopy. (This figure is available in color at *JXB* online.)

To analyse the subcellular distribution of CIPK8, its open reading frame fused with the GFP gene was inserted into the plasmid pCAMBIA1300. SOS2, a cytoplasm-localized protein (Kim *et al.*, 2007), was used as a control. GFP-tagged SOS2, CIPK8, or GFP alone was transiently expressed in Arabidopsis protoplasts. When GFP by itself is expressed in plant cells, the protein can be seen in the cytoplasm under a fluorescence microscope (Zhou *et al.*, 2016), and the fluorescence images of the GFP-tagged SOS2 protein was similar to the typical cytosolic fluorescence signal distribution of GFP (Supplementary Fig. S3), suggesting that the SOS2 protein localizes to the cytoplasm. Like GFP alone or SOS2–GFP, CIPK8–GFP also exhibited a diffused fluorescence pattern in the plant cells, indicating that CIPK8 might make the same contribution in cytoplasm as SOS2. To further explore whether CIPK8 and SOS2 localize to the same cellular compartment in plant cells, two fusion constructs, *CIPK8-YFP* (yellow fluorescent protein) and *SOS2-CFP* (cyan fluorescent protein), were co-transformed into tobacco leaf cells and Arabidopsis mesophyll protoplasts. The fluorescence signals in the transformed tobacco leaf cells and Arabidopsis mesophyll protoplasts were detected using a confocal laser scanning microscope. The fluorescence patterns of CIPK8 and SOS2 overlapped clearly with the same localization pattern, indicating a high degree of co-localization between them (Fig. 1). Arabidopsis SOS1 consists of a transmembrane N-terminus and a cytoplasmic C-terminus, the latter of which contains important regulatory domains. SOS2 interacts with the cytoplasmic C-terminal tail of SOS1 and regulates the salt tolerance capability of Arabidopsis plants (Quintero *et al.*, 2011). Since CIPK8 and SOS1 co-localize to cytoplasm, it is possible that CIPK8 may regulate SOS1 activity by interacting with its C-terminal cytoplasmic tail, analogous to SOS2.

### Expression pattern of CIPK8

**Fig. 2.**
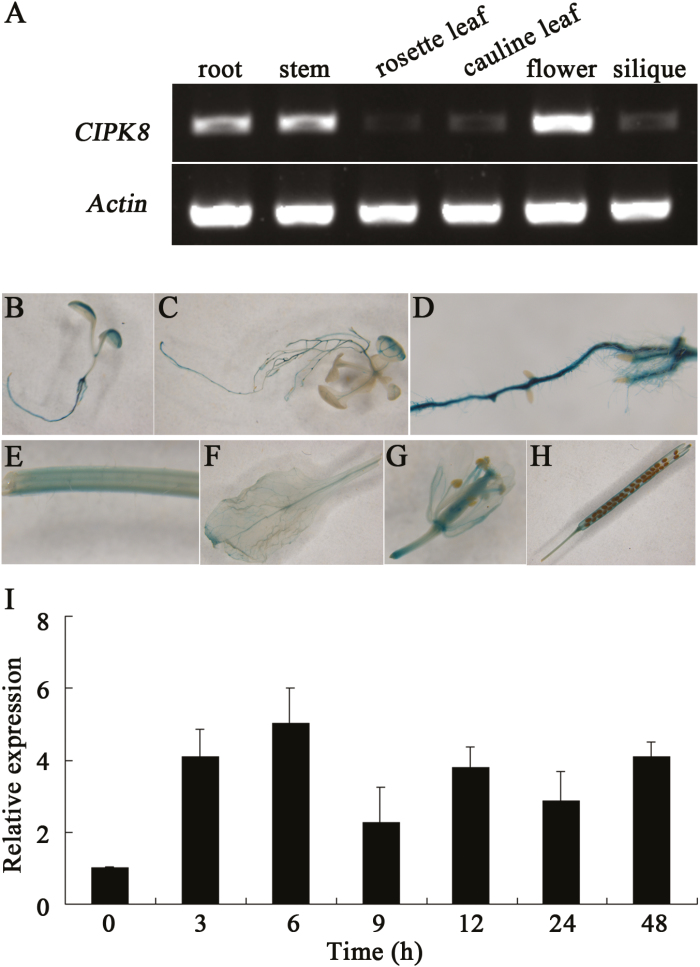
Expression of *CIPK8* in Arabidopsis. (A) Expression of *CIPK8* in the roots, stems, rosette leaves, cauline leaves, flowers, and siliques. RT-PCR was performed to determine *CIPK8* expression in different tissues with gene-specific primers. *CIPK8* levels were normalized to *actin*. (B–H) *CIPK8* promoter-*GUS* expression in the early seedling cotyledons (B), seedling rosette leaves (C), roots (D), stems (E), leaves (F), flowers (G), and siliques (H). (I) Expression of *CIPK8* in Arabidopsis seedlings treated with 100 mM NaCl. *CIPK8* expression was analysed by qRT-PCR with gene-specific primers. *CIPK8* levels were normalized to *actin*. Data represent the mean ±SE of three replicates. (This figure is available in color at *JXB* online.)

The amount of *CIPK8* transcript present in different tissues was measured by RT-PCR. The results indicated that the gene was expressed in all of the tissues tested, but differential expression patterns across the tissues tested revealed that *CIPK8* is highly expressed in the roots, stems, and flowers, but was very lowly expressed in the siliques (Fig. 2A). To confirm the expression pattern of the gene, the promoter region of *CIPK8* was combined with a *GUS* (β-glucuronidase) reporter gene in the plasmid pCAMBIA1301. This construct was introduced into Arabidopsis, and transgenic plants were analysed with the GUS staining method. *CIPK8* is expressed in the entire plant, but consistent with our RT-PCR results, the *GUS* expression level in roots, stems, and flowers driven by the *CIPK8* promoter was higher than that in the other tissues tested (Fig. 2B–H). The expression of *CIPK8* in Arabidopsis seedlings exposed to 100 mM NaCl was analysed using RT-PCR. Upon introduction of salinity stress, the transcript level of *CIPK8* increased initially and then decreased slightly, but the final mRNA abundance 48 h after NaCl treatment was approximately 4-fold higher than that at 0 h (Fig. 2I). *SOS2* is reported to be expressed throughout the entire plants, and its expression level is also induced by salt treatment (Quan *et al.*, 2007). The similar expression pattern of *SOS2* and *CIPK8* suggests that CIPK8 may function in the SOS pathway, analogous to SOS2.

### The response of *cipk8* and *sos2* to salinity stress

**Fig. 3.**
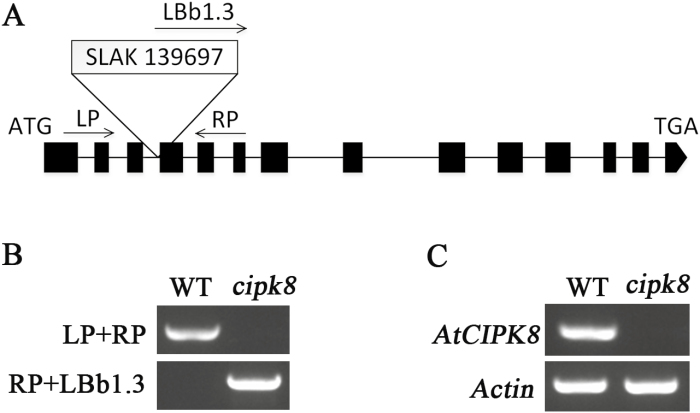
Characterization of *CIPK8* mutant. (A) Schematic diagram of *CIPK8* and T-DNA position in the *CIPK8* genomic sequence. SALK_139697: the number of *cipk8* in the Arabidopsis mutant library. *CIPK8*-specific primers (LP, RP) and a T-DNA left border primer (LBb1.3) were used to identify a T-DNA insertion in the *CIPK8* gene. (B) Confirmation of presence of the *cipk8* mutation. PCR was performed with primers described in Supplementary Table S1 and using template genomic DNA from either *cipk8* or wild-type plants. (C) *CIPK8* expression assays in *cipk8* and wild-type plants by RT-PCR. Gene expression levels were normalized to *actin*. RT-PCR primers for *CIPK8* are described in Supplementary Table S1.

**Fig. 4.**
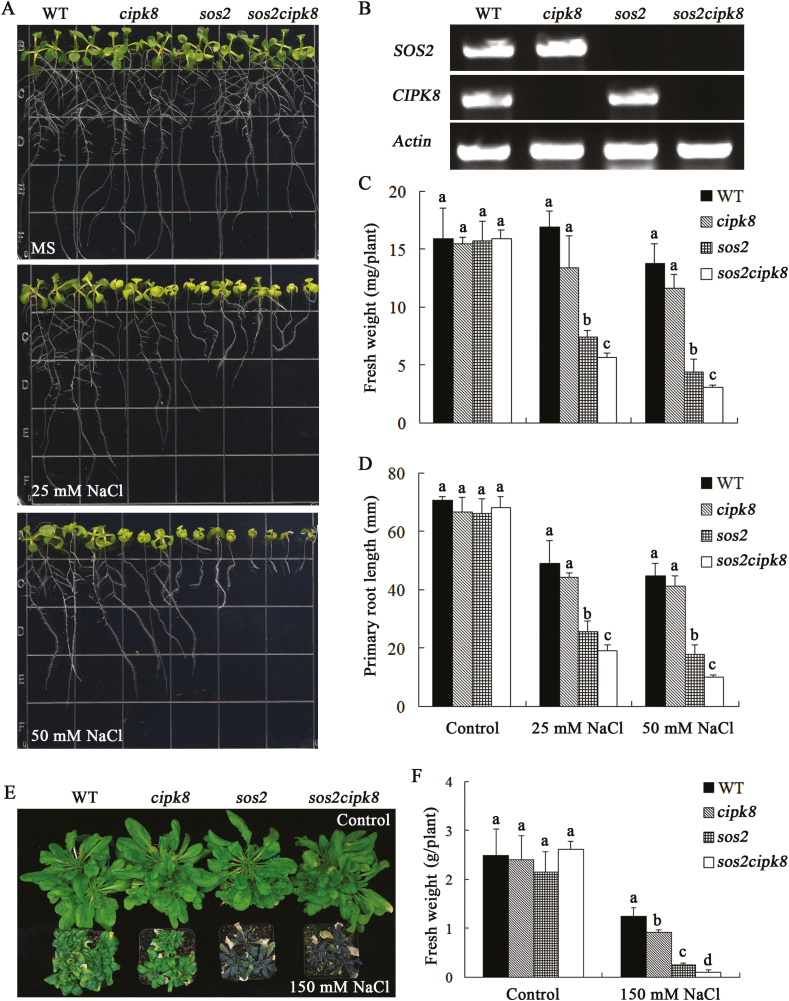
Effects of NaCl treatment on the growth of WT, *cipk8*, *sos2*, and *sos2cipk8* plants. (A) Images of WT, *cipk8*, *sos2*, and *sos2 cipk8* seedlings grown under conditions of salt stress for 7 d. (B) Expression levels of *SOS2* and *CIPK8* in WT, *cipk8*, *sos2*, and *sos2cipk8* plants by RT-PCR (primers listed in Supplementary Table S1). Gene expression levels were normalized to *actin*. (C) Fresh weight of WT, *cipk8*, *sos2*, and *sos2cipk8* seedlings measured after 7 d of growth under salt stress conditions. Data represent the mean ±SE of 12 replicates. (D) Primary root lengths of WT, *cipk8*, *sos2*, and *sos2cipk8* seedlings measured after 7 d of growth in salt stress conditions. Data represent the mean ±SE of 12 replicates. (E) WT, *cipk8*, *sos2*, and *sos2cipk8* plants were allowed to grow in normal soil for 3 weeks, and were then grown in soil treated with 150 mM NaCl for 4 weeks. The images were taken after 4 weeks of NaCl treatment. (F) After growing in soil treated with 150 mM NaCl for 4 weeks, the fresh weights of WT, *cipk8*, *sos2*, and *sos2cipk8* plants were determined. Data represent the mean ±SE of nine replicates. Letters above the columns indicate significant differences with *P*<0.05. (This figure is available in color at *JXB* online.)

The T-DNA insertion mutant of *CIPK8* (*cipk8*) from ABRC (SALK_139697) was used to investigate the salt tolerance phenotype of CIPK8 in Arabidopsis. The localization of T-DNA to the fourth exon of the *CIPK8* gene was confirmed by PCR with the gene-specific primers and a T-DNA left border primer (Fig. 3A, B). The T-DNA insertion completely abrogated expression of the *CIPK8* gene in *cipk8* plants (Fig. 3C). *cipk8*, *sos2*, and wild-type seedlings were grown on MS plates for 5 d, and were then transferred onto MS plates containing 50 mM NaCl. The three plant strains could grow in normal MS medium, but the growth of *cipk8* was slightly inhibited by the NaCl treatment relative to wild-type seedlings, and *sos2* displayed the most significant growth reduction under salinity stress (see Supplementary Fig. S4). To further investigate the role of CIPK8 in promoting salt tolerance, the double knockout mutant *sos2cipk8* was generated from the *sos2* and *cipk8* mutants. The transcripts of *CIPK8* and *SOS2* were not detectable in *sos2cipk8* by RT-PCR (Fig. 4B). The absence of *CIPK8* and *SOS2* made the double mutant highly sensitive to salt stress, and the fresh weight of *sos2cipk8* was 30% less than that of the single knockout *sos2* grown on MS plates containing 50 mM NaCl (Fig. 4A–D).

To accurately assess the plant salt tolerance phenotype, 10-day-old seedlings were transferred from MS plates into normal soil and grown for 3 weeks, and were then treated with 150 mM NaCl for 4 weeks. After that, the plants were photographed and weighed. The fresh weight of *cipk8* was greater than that of *sos2* after NaCl treatment, but the growth of *cipk8* was more significantly inhibited by salt treatment compared with wild-type plants. Furthermore, the *sos2cipk8* double mutant showed severely diminished growth relative to *sos2* or *cipk8* under conditions of salinity stress. The double mutant was so severely affected by the saline conditions that some of its leaves became bleached (Fig. 4E, F).

### Complementation to salt sensitivity of *cipk8* mutant

**Fig. 5.**
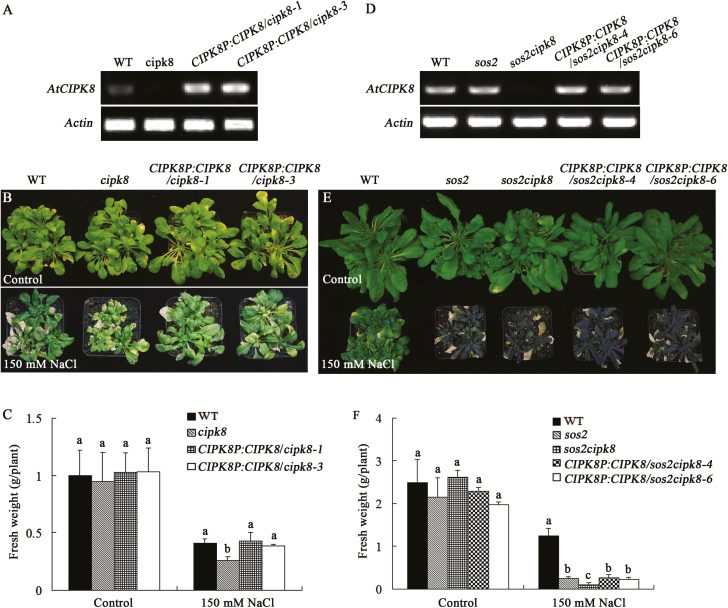
CIPK8 complements salt sensitivity of *cipk8* and *cipk8sos2*. A 5502 bp DNA fragment containing the promoter and coding region of *CIPK8* (*CIPK8P*:*CIPK8*) was introduced into *cipk8* or *sos2cipk8* mutants through mediation of the vector pCAMBIA1300. The T3 generations of 10 transgenic homozygote lines of *CIPK8* in *cipk8* (*CIPK8P*:*CIPK8*/*cipk8*) and eight transgenic homozygote lines of *CIPK8* in *sos2cipk8* (*CIPK8P:CIPK8*/*sos2cipk8*) were used for screening. The transgenic lines, *CIPK8P:CIPK8*/*cipk8*-1, *CIPK8P:CIPK8*/*cipk8*-3, *CIPK8P:CIPK8*/*sos2cipk8*-4 and *CIPK8P:CIPK8*/*sos2cipk8*-6, were used for the following experiments. (A) *CIPK8* expression levels in WT, *cipk8*, and *CIPK8P:CIPK8*/*cipk8* lines were determined by RT-PCR with gene-specific primers (Supplementary Table S1). (B) WT, *cipk8*, and *CIPK8P:CIPK8*/*cipk8* plants were grown in normal soil for 3 weeks, and then grown for 4 weeks in soil supplemented with 150 mM NaCl. Images were taken after 4 weeks of NaCl treatment and the fresh weights (C) were determined. (D) *CIPK8* expression levels in WT, *sos2*, *sos2cipk8*, and *CIPK8P:CIPK8*/*sos2cipk8* lines were determined by RT-PCR with gene-specific primers (Supplementary Table S1). (E) WT, *sos2*, *sos2cipk8*, and *CIPK8P:CIPK8*/*sos2cipk8* plants were grown in normal soil for 3 weeks, and then grown for 4 weeks in soil supplemented with 150 mM NaCl. Images were taken after 4 weeks of NaCl treatment, and the fresh weights (F) were determined. Data represent the mean ±SE of nine replicates and letters above the columns indicate significant differences with *P*<0.05. (This figure is available in color at *JXB* online.)

A 5502 bp DNA fragment corresponding to the genomic sequence from 2399 bp upstream of the start codon of *CIPK8* was cloned by PCR according to the sequence of the Arabidopsis genome and then inserted into the plasmid pCAMBIA1300. The resulting construct was introduced into *cipk8* and *sos2cipk8*. The transcription of *CIPK8* was restored in double mutant *sos2cipk8* plants expressing the construct (Fig. 5A, D), and the salt sensitivity of *sos2cipk8* was partially rescued. However the double mutant transformed with *CIPK8* displayed a similar growth pattern to the *sos2* single mutant, rather than the wild-type plants, after salt treatment (Fig. 5E, F; Supplementary Fig. S5). Interestingly, *CIPK8* transformation in *cipk8* completely restored the salt tolerance of the *cipk8* mutant. The growth of the *cipk8* mutant re-expressing *CIPK8* was similar with that of wild-type plants under conditions of salt stress (Fig. 5B, C). These results suggest that re-expressing *CIPK8* is sufficient to at least partially restore the salt tolerance capability of *cipk8* or *sos2cipk8*, but its function may be less crucial for regulating the Arabidopsis response to salt stress relative to SOS2.

### The interactions between CIPK8 and CBLs

**Fig. 6.**
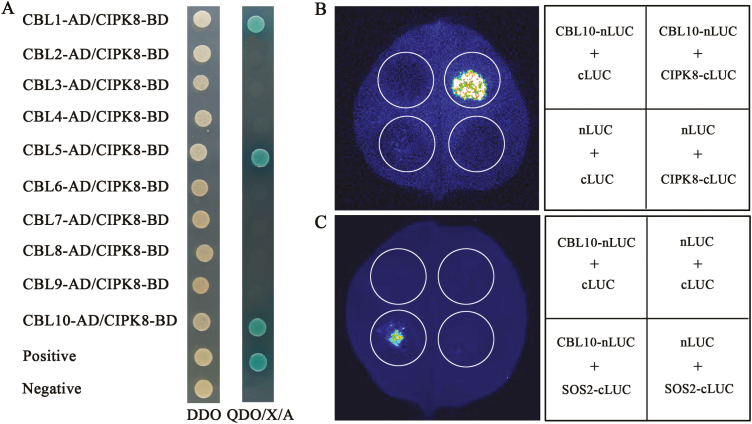
Identifying interactions between CIPK8 and CBL proteins. (A) Interaction between CIPK8 and CBL proteins was assayed using a yeast two-hybrid method. Yeast cells harboring different fusion protein combinations were plated on DDO medium (lacking Leu and Trp) or QDO/X/A (lacking Leu, Ade, His, and Trp, but supplemented with 40 µg ml^−1^ X-α-Gal and 125 ng ml^−1^ aureobasidin A). The combination pGADT7-T and pGBKT7-53 was used as a positive control, and pGADT7-T and pGBKT7-lam was used as a negative control. (B) Interactions between CIPK8 with CBL10 determined using firefly luciferase complementation imaging assays in *Nicotiana benthamiana* leaves. The interaction between SOS2 and CBL10 was used as positive conterol (C). cLUC, C-terminal region of firely luciferase; nLUC, N-terminal region of firefly luciferase. Three independent experiments were carried out in this study. (This figure is available in color at *JXB* online.)

The salt-sensitive phenotype of the *sos2cipk8* double mutant was more severe than *sos2* or *cipk8* (Fig. 4), but its salt-sensitive phenotype was quite similar to *sos1* (see Supplementary Fig. S6), suggesting that CIPK8 and SOS2 may coordinate to regulate the Na^+^/H^+^ exchange activity of SOS1. The SOS signaling pathway was initially defined by three components, SOS1, SOS2, and SOS3, with both SOS2 and SOS3 required for the activation of the SOS1 Na^+^/H^+^ exchange activity, indicating that the regulatory effect of SOS2 on SOS1 is SOS3-dependent (Qiu *et al.*, 2002; Quintero *et al.*, 2002; Quintero *et al.*, 2011). In a transgenic yeast mutant strain lacking the plasma membrane Na^+^ transporter, no growth difference was observed between yeast cells co-expressing *CIPK8* and *SOS1* and single transgenic cells with *SOS1* when grew on AP plates supplemented with NaCl (Supplementary Fig. S7), suggesting that the regulation of CIPK8 on SOS1 may be CBL-dependent. To find the putative CBL regulator of CIPK8, interaction between each of the CBL proteins and CIPK8 was assayed using the yeast two-hybrid method. Surprisingly, yeast cells transformed with SOS3 and CIPK8 did not grow on the screening plate. The CBL family has 10 members in Arabidopsis, but we found that only three, CBL1, CBL5, and CBL10, interacted with CIPK8 (Fig. 6A). To confirm whether the three pairs of CBLs–CIPK8 identified from the yeast two-hybrid method can interact in plant cells *in vivo*, we performed interaction assays between CBL1 and CIPK8, CBL5 and CIPK8, and CBL10 and CIPK8 using the Split-LUC technique. Since it has been previously reported that CBL10 can interact with SOS2 in Arabidopsis cells (Quan *et al.*, 2007), and since no interaction signal between SOS3 and CIPK8 was detected in the yeast two-hybrid analysis (Fig. 6A), the interactions between CBL10 and SOS2, and SOS3 and CIPK8 was used as positive and negative controls, respectively. As expected, no fluorescence signal was detected for the combination of SOS3 and CIPK8 (data not shown). By contrast, a robust fluorescence signal was very clear for cells co-transfected with CBL10 and SOS2. The fluorescence signal for the combinations of CBL1, CBL5, or CBL10 with CIPK8 was similar to that of the CBL10–SOS2 combination in tobacco leaf cells (data not shown; Fig. 6B). These results indicate that CIPK8 can directly interact with CBL1, CBL5, and CBL10.

### Regulation of CBLs–CIPK8 complexes on SOS1 function in yeast cells

**Fig. 7.**
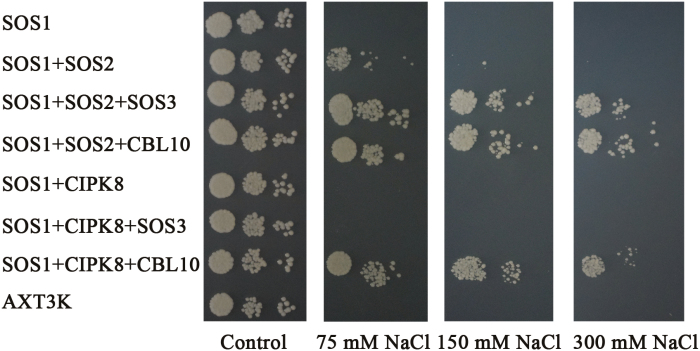
CIPK8 regulation of SOS1 activity in yeast cells. *SOS1* and *CIPK8*/*SOS2* were cloned into the plasmids p416 (p416-*SOS1*) and p414 (p414-*CIPK8*, p414-*SOS2*), respectively; *CIPK8/SOS2* and *SOS3* or *CIPK8/SOS2* and *CBL10* were cloned into p414 (p414-*CIPK8-SOS3*, p414-*CIPK8-CBL10*, p414-*SOS2-SOS3*, and p414-*SOS2-CBL10*) as described in ‘Materials and methods’. p416-*SOS1* alone or in other indicated combinations was transformed into AXT3K. Transgenic and untransformed yeast cells were spotted on AP plates with or without NaCl, as described in ‘Materials and methods’, and cultured at 28 °C for 3–5 d. SOS1, p416-*SOS1*; SOS2, p414-*SOS2*; SOS2+SOS3, p414-*SOS2*-*SOS3*; SOS2+CBL10, p414-*SOS2*-*CBL10*; CIPK8, p414-*CIPK8*; CIPK8+SOS3, p414-*CIPK8*-*SOS3*;CIPK8+CBL10, p414-*CIPK8*-*CBL10*.

SOS3/CBL10–SOS2 complexes can phosphorylate and activate the Na^+^/H^+^ exchange activity of SOS1, thereby mediating Na^+^ extrusion under conditions of salinity stress. The functions of these two arms of the SOS pathway have been determined using the yeast mutant strain AXT3K, which lacks plasma membrane Na^+^ transporters (Martínez-Atienza *et al.*, 2007; Quan *et al.*, 2007; Quintero *et al.*, 2011). To investigate the regulation effects of the CBLs–CIPK8 complexes (CBL1–CIPK8, CBL5–CIPK8, and CBL10–CIPK8) on SOS1 activity, three groups of genes (*CBL1*, *CIPK8*, and *SOS1*; *CBL5*, *CIPK8*, and *SOS1*; and *CBL10*, *CIPK8*, and *SOS1*) were separately transformed into AXT3K. The two known SOS pathways, SOS3–SOS2–SOS1 and CBL10–SOS2–SOS1, were used as positive controls. In addition, the response of AXT3K transformed with *SOS3*, *CIPK8*, and *SOS1* to salt stress was also tested because our yeast two-hybrid and bimolecular complementation assays did not detect an interaction between SOS3 and CIPK8 (Fig. 6). The yeast mutant strain AXT3K was sensitive to salt treatment and could not survive in AP medium supplemented with 75 mM NaCl. SOS1 slightly rescued the salt sensitivity phenotype of the mutant strain. Co-expression of *SOS1* and *CIPK8* was unable to improve the growth of transgenic yeast cells relative to the transformant with *SOS1* alone under conditions of salt stress (Supplementary Figs S7, S8; Fig. 7). Unexpectedly, although CBL1 can interact with CIPK8 (Fig. 6), yeast cells transformed with *CBL1*, *CIPK8*, and *SOS1* displayed similar growth to yeast cells expressing *SOS1* alone, suggesting that the salt stress function of SOS1 is not activated by the CBL1–CIPK8 complex (see Supplementary Fig. S8). In yeast, the SOS3/CBL10–SOS2 complexes upregulate SOS1 activity, such that concurrent expression of these *SOS* genes significantly boosts the salt tolerance ability of transgenic yeast cells, which could grow in AP medium containing a high concentration of NaCl (Fig. 7; Quintero *et al.*, 2002, 2011; Martínez-Atienza *et al.*, 2007; Quan *et al.*, 2007). Co-expression of CBL10 and CIPK8 in the presence of SOS1 greatly enhanced the salt tolerance of transgenic yeast to a similarly high level to that achieved with co-expression of the four reported SOS components (CBL10/SOS3, SOS2 and SOS1) (Fig. 7). This increased salt tolerance phenotype was observed only when CBL10 was present, suggesting that CIPK8-mediated activation of SOS1 is CBL10-dependent. It has been reported that the growth of *cbl10* is severely inhibited by salt treatment, and *CBL10* expression can complement salt sensitivity of the mutant (Quan *et al.*, 2007). In the branch of the SOS pathway composed of CBL10, CIPK8, and SOS1, loss of function mutations in these three genes results in increased sensitivity to NaCl treatment (Quan *et al.*, 2007, Figs 4, 5; Supplementary Fig. S1), indicating that the CBL10–CIPK8–SOS1 pathway is involved in promoting the salt tolerance capability of Arabidopsis.

### CBL10–CIPK8 complex activates SOS1 Na^+^/H^+^ antiport activity

**Fig. 8.**
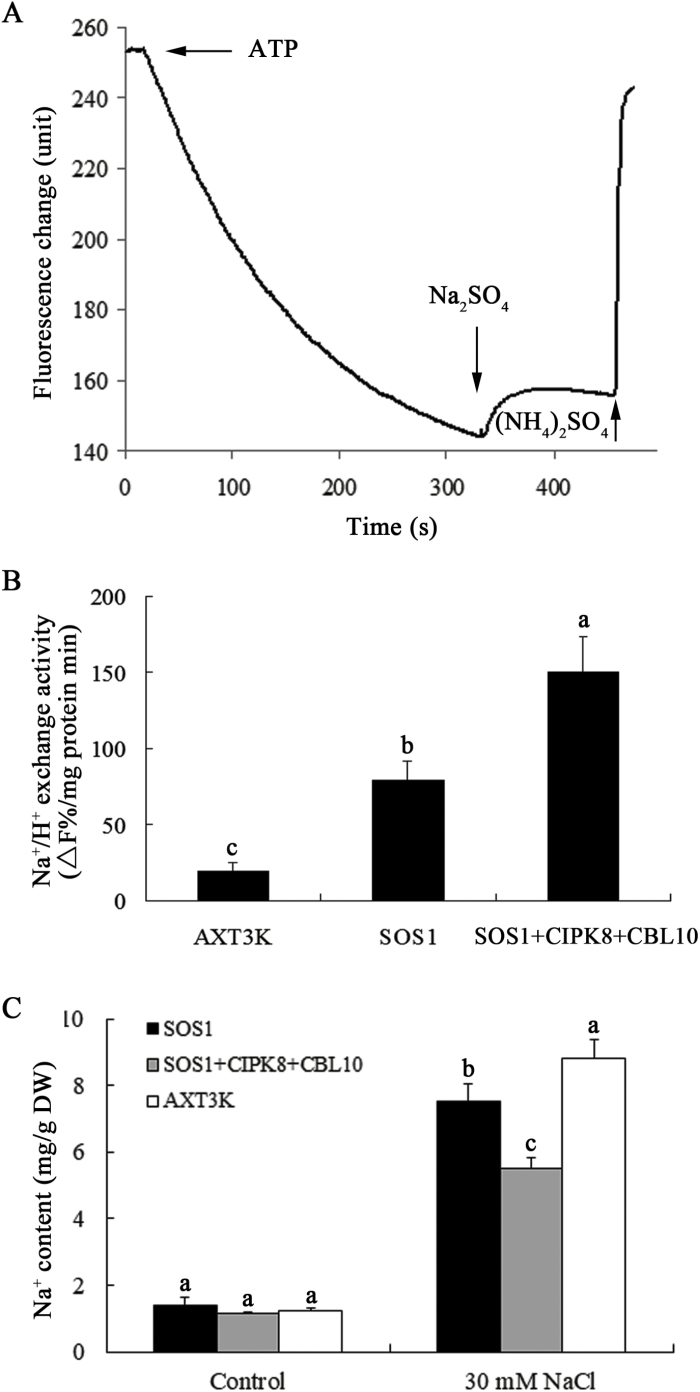
SOS1-mediated regulation of CBL10 and CIPK8 promotes Na^+^/H^+^ exchange. Plasma membranes were isolated from mutant cells (AXT3K) and related transgenic strains (*SOS1* or *SOS1+CIPK8+CBL10*) using the aqueous two-phase system. (A) Na^+^/H^+^ exchange activity in plasma membrane vesicles. A pH gradient was established using ATP, indicating that protons were pumped into the vesicles. Once the fluorescence signal stabilized, 50 mM Na_2_SO_4_ was added to the cuvette, and fluorescence recovery, indicating active Na^+^/H^+^ exchange, was monitored. The reaction was terminated by adding 25 mM (NH_4_)_2_SO_4_, which dissipated the pH gradient. The change in fluorescence is expressed in arbitrary units. One representative experiment is shown. (B) Na^+^/H^+^ exchange activity given as the proportion of dissipation of the established pH gradient per minute per milligram of membrane protein (Δ*F*% min^−1^ mg protein^−1^). (C) Total Na^+^ content in untransformed and transgenic yeast cells. Data are expressed as the means ±SE of three replicates and letters above the columns indicate significant differences with *P*<0.05.

The Na^+^/H^+^ exchange activity of Arabidopsis SOS1 has been detected in the plasma membrane of transgenic yeast cells with *SOS1* (Martínez-Atienza *et al.*, 2007; Xu *et al.*, 2008). To determine whether CBL10 and CIPK8 regulate the Na^+^/H^+^ exchange activity of SOS1, *CBL10*, *CIPK8*, or *SOS1* was introduced into the yeast mutant strain AXT3K alone or in combination. Once yeast cells grew to a suitable optical density in AP medium, 100 mM NaCl was added to the medium and the culture was grown for another 1 h to ensure activation of SOS1 by the CBL10–CIPK8 kinase complex when present, as previously reported (Martínez-Atienza *et al.*, 2007). After 1 h, the plasma membrane vesicles were isolated from the yeast cells, and plasma membrane Na^+^/H^+^ exchange activity was determined using the quinacrine fluorescence quenching method (Fig. 8A). The Na^+^/H^+^ exchange activity of plasma membrane vesicles from yeast cells co-expressing *SOS1*, *CBL10*, and *CIPK8* was the highest, followed by the activity of yeast cells expressing *SOS1* alone (Fig. 8B). Minimal Na^+^/H^+^ exchange activity was observed across the plasma membrane of untransformed yeast cells. This low plasma membrane Na^+^/H^+^ antiport activity led to significant Na^+^ accumulation in AXT3K cells treated with NaCl. As expected, the sodium content of transgenic yeast cells co-expressing *CBL10*, *CIPK8*, and *SOS1* was the lowest of all combinations tested, and the intracellular Na^+^ concentration was 38% and 27% lower than that of untransformed and *SOS1*-expressing cells, respectively (Fig. 8C). These results indicate that the CBL10–CIPK8 complex positively regulates SOS1 Na^+^/H^+^ antiporter activity, and the CBL10–CIPK8–SOS1 pathway can efficiently promote the transport of excess Na^+^ out of yeast cells under conditions of salt stress.

### Analysis of Na^+^ effluxes in leaves of wild-type and *cipk8* mutant plants

**Fig. 9.**
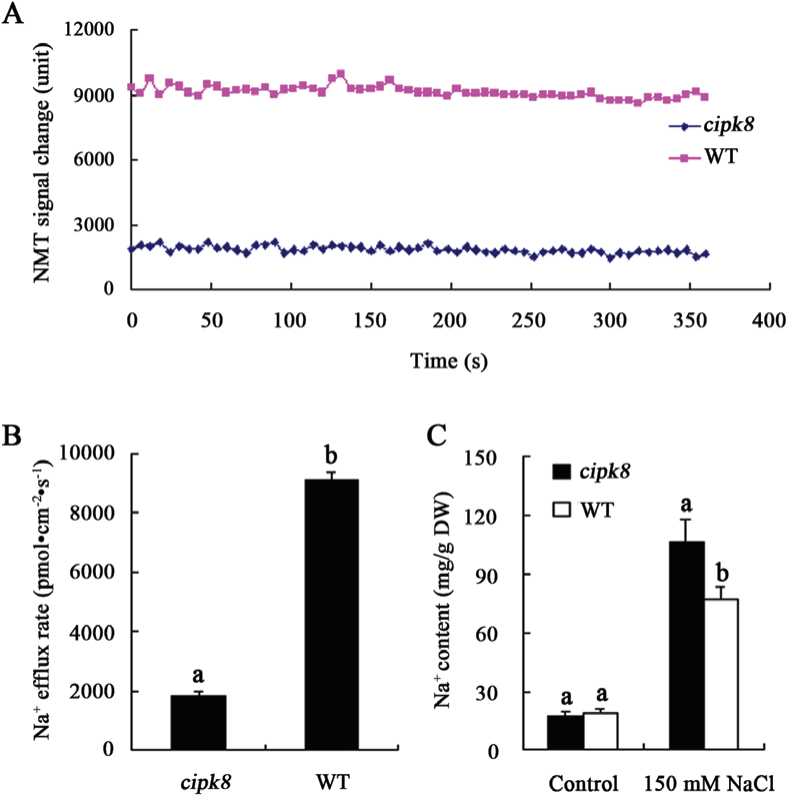
Na^+^ flux in leaves and total Na^+^ content in wild-type and *cipk8* mutant plants. (A) The change in NMT signal is expressed as arbitrary units. Seedlings were grown in MS plates containing 100 mM NaCl for 24 hours, after which Na^+^ flux in leaves was measured using the NMT technique as described in ‘Materials and methods’. (B) Na^+^ efflux activity is measured as the concentration of exported Na^+^ per second per square centimeter (pmol cm^−2^ s^−1^), and data represent the mean ±SE of six replicates. (C) Changes in the total Na^+^ content in *cipk8* and wild-type leaves under conditions of salt stress; data represent the mean ±SE of three replicates. Letters above the columns indicate significant differences with *P*<0.05. (This figure is available in color at *JXB* online.)

It has been reported that in the absence of environmental stressors, SOS1 is kept in a resting state by a C-terminal auto-inhibitory domain that interacts with and occludes the adjacent activation domain essential for SOS1 activity. Upon introduction of salinity stress, the Ca^2+^-dependent SOS2–SOS3 protein kinase complex phosphorylates SOS1 and relieves SOS1 auto-inhibition, presumably by displacing the auto-inhibitory C-terminal domain. Activated SOS1 then transports Na^+^ out of cells using the energy from the proton gradient established by the H^+^-ATPase (Quintero *et al.*, 2011). In this scenario, the Na^+^/H^+^ exchange activity of SOS1 could be regulated by CIPK8 in the co-transgenic yeast cells (Fig. 8). We therefore hypothesized that the salt tolerance mechanism regulated by CIPK8 may be involved in promoting SOS1-mediated Na^+^ transport out of plant cells. To directly test this hypothesis, Na^+^ flux in leaves was analysed using the NMT (non-invasive micro-test technology) technique. All leaves from WT and *cipk8* mutant plants treated with NaCl displayed Na^+^ efflux characteristics. Comparative analyses showed that the Na^+^ efflux rate in wild-type leaves was 5-fold faster than that of the *cipk8* mutant leaves under salt stress conditions (Fig. 9A, B). These differences in transport rate resulted in lower Na^+^ content in the wild-type leaves compared with that in the *cipk8* mutant leaves (Fig. 9C), indicating that the reduced Na^+^ extrusion might be a phenotype of the *cipk8* mutant. SOS1 is the only plasma membrane protein with Na^+^ export activity characterized to date (Quintero *et al.*, 2011). Therefore, these findings further suggest that the new SOS pathway identified here, CBL10–CIPK8–SOS1, is involved in regulating plant salt tolerance by promoting Na^+^ export from cells.

### Identification of CBL10-CIPK8 regulatory sites in the SOS1 sequence

**Fig. 10.**
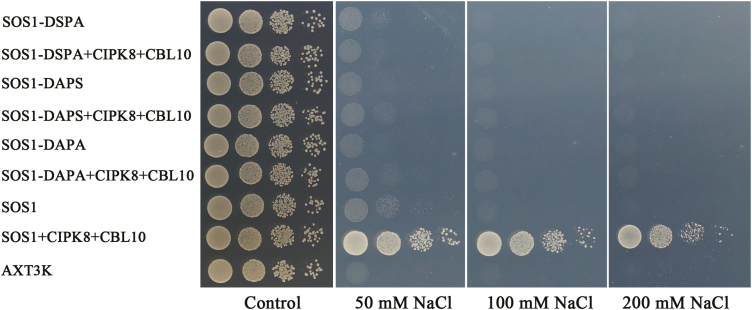
Identification of sites in the SOS1 sequence regulated by the CIPK8–CBL10 complex. Site-specific mutations in SOS1 were generated at the 1136th and 1138th serine residues as described in ‘Materials and methods’. The wild-type and mutant *SOS1* genes (wild-type gene (DSPS), mutant S1136A (DAPS), mutant S1138A (DSPA), and double mutant S1136A/S1138A (DAPA)) were cloned into the vector p416, and transformed into AXT3K either lacking or co-expressing *CIPK8* and *CBL10*. Transgenic and untransformed yeast cells were spotted on AP plates with or without NaCl as described in ‘Materials and methods’, and cultured at 28 °C for 3–5 d.

The SOS1 protein consists of a transmembrane N-terminus and a C-terminus containing several key regulatory domains. The SOS3–SOS2 protein kinase complex is known to phosphorylate SOS1 at serine 1138 (S1138) in the C-terminus, which regulates SOS1 Na^+^/H^+^ exchange activity. While serine 1136 (S1136) is not the phosphorylation site, it is critical for helping localize SOS2 to the phosphorylation site. Therefore, S1136 and S1138 on SOS1 are essential for SOS3–SOS2-mediated activation (Quintero *et al.*, 2011). To determine whether CBL10–CIPK8 can regulate SOS1 activity through the same sites, S1136 and S1138 were mutated to alanine either individually (S1136A, S1138A) or together (S1136AS1138A) by site-directed mutagenesis. Then *CBL10*, *CIPK8*, and the three *SOS1* mutant genes (SOS1-S1136A, SOS1-S1138A, and SOS1-S1136AS1138A) were separately introduced into AXT3K. The S1136A, S1138A, and S1136AS1138A mutations all completely abrogated the regulatory effects of CBL10–CIPK8 on SOS1 activity in AXT3K grown in AP medium supplemented with NaCl (Fig. 10). This finding is consistent with the regulatory roles of SOS3 and SOS2 on SOS1 function (Quintero *et al.*, 2011), suggesting that CBL10–CIPK8 similarly activates SOS1 by phosphorylating the same regulatory sites (S1136 and S1138) at the SOS1 C-terminus when Arabidopsis is exposed to conditions of increased salinity.

## Discussion

Sodium is a trace element in plant and excessive cytoplasmic Na^+^ can disrupt a variety of physiological and biochemical processes, which can be quite toxic to both cells and the whole plant. Therefore, the efficient extrusion of Na^+^ from cells plays a key role in helping plants adapt to and survive in a saline environment. SOS1, a plasma membrane Na^+^/H^+^ antiporter, is known to mediate Na^+^ extrusion from cells. Sodium accumulation in the *sos1* mutant resulted in reduced growth under salinity stress (Wu *et al.*, 1996; Zhu *et al.*, 1998; Oh *et al.*, 2009); the reduction of *SOS1* expression resulted in excessive accumulation of Na^+^ in RNAi tomato plants and a loss of the salt tolerance phenotype (Oliás *et al.*, 2009). In contrast, plants overexpressing *SOS1* displayed lower intracellular Na^+^ levels and better growth compared with wild-type plants upon NaCl treatment (Zhou *et al.*, 2016). The Na^+^/H^+^ exchange activity of SOS1 has been characterized using proteoliposomes and plasma membrane vesicles isolated from yeast cells expressing the *SOS1* gene (Martínez-Atienza *et al.*, 2007; Xu *et al.*, 2008; Quintero *et al.*, 2011; Núñez-Ramírez *et al.*, 2012). While SOS1 is involved in promoting Na^+^ efflux from cells and plant salt tolerance, its Na^+^/H^+^ exchange activity is inhibited in plants when grown under normal environmental conditions (Quintero *et al.*, 2011; Zhou *et al.*, 2016). A variety of additional mechanisms are known to play important roles in regulating SOS1 activity under various stress conditions. When plants are affected by salt stress conditions, the level of calcium in the cytosol spikes (Knight, 2000). SOS3 acts as a calcium sensor and responds to the change in calcium signal triggered by salt stress. When Ca^2+^ levels increase, SOS3 binds to Ca^2+^ and is then able to interact with SOS2 to form an activated complex that positively regulates SOS1 activity in roots (Shi *et al.*, 2000; Qiu *et al.*, 2002; Quintero *et al.*, 2002; Martínez-Atienza *et al.*, 2007; Tang *et al.*, 2010). Quan *et al.* (2007) reported that CBL10 interacts with and recruits SOS2 to the plasma membrane to activate the Na^+^/H^+^ exchange activity of SOS1 in shoots. A regulator of the cellular oxidative stress response, radical-induced cell death (RCD1), interacts with SOS1 and regulates the response of Arabidopsis to salt and oxidative stress (Katiyar-Agarwal *et al.*, 2006). Phospholipase D (PLD) hydrolyses phospholipids to produce phosphatidic acid (PA). When Arabidopsis plants were exposed to salinity stress, PA and phospholipase Dα1 (PLDα1) regulated the activity of mitogen-activated protein kinase 6 (MPK6), and the activated MPK6 then simulated SOS1 activity. Both *mpk6* and *pldα1* mutants demonstrated increased Na^+^ accumulation and salt sensitivity compared with wild-type plants (Yu *et al.*, 2010), indicating that the PLDα1–PA–MPK6–SOS1 signaling system is a key regulatory pathway for cellular Na^+^ exclusion and promotion of salt tolerance of Arabidopsis. The salt tolerance of *cbl10cipk24* was lower relative to *cipk24*, indicating that CBL10 might regulate the response of Arabidopsis plants to salinity via interactions with other partners besides CIPK24 (Yang *et al.* 2019). CIPK8 is the closest homolog to CIPK24 among the 26 CIPK members, and in the present study, CIPK8 was found to interact with CBL1, CBL5, and CBL10 (Fig. 6A), but only co-expression of *CBL5* with *CIPK8* or *CBL10* with *CIPK8* could improve the growth of *SOS1*-transgenic yeast cells treated with NaCl (see Supplementary Fig. S8). Zhang *et al.* (2013) found that CBL1 (PeCBL1) can interact with CIPK24 (PeCIPK24) in *Populus euphratiica*, and Arabidopsis overexpressing *PeCBL1* exhibited decreased growth compared with wild-type plants under salt stress, indicating that PeCBL1 might negatively regulate the response of *P. euphratiica* plants to salinity stress through interacting with PeCIPK24, which is consistent with our results that CBL1 and CIPK8 from Arabidopsis plants interact with each other but could not further activate the function of SOS1. Although CBL5 and CIPK8 could also positively regulate SOS1 activity in yeast cells exposed to salinity stress similarly to CBL10 and CIPK8 (Supplementary Fig. S8), both *cipk8* and *sos1* mutants were sensitive to salt stress while the *cbl5* mutant did not display a noticeable salt-sensitive phenotype relative to wild-type Arabidopsis plants (data not shown), so the mechanistic relationship between the components of the CBL5–CIPK8–SOS1 pathway and plant salt tolerance remains unclear. Our results establish that the plasma membrane Na^+^/H^+^ antiporter SOS1 is a downstream target of the CBL10–CIPK8 complex. The Na^+^ efflux from *cipk8* mutant cells was slower than that from wild-type cells under saline stress, resulting in more Na^+^ accumulation in *cipk8* mutant plants (Fig. 9), which therefore exhibited a worse growth phenotype relative to wild-type plants under saline stress (Fig. 4E). In addition, complementation testing showed that *CIPK8* expression could rescue the inhibited growth phenotype of *cipk8* knockout plants under salt stress (Fig. 5). These results indicate that loss of function of CIPK8 is responsible for the reduced Na^+^ extrusion and decreased growth. Thus, the association among reduced Na^+^ extrusion, higher intracellular sodium level and the salt sensitivity of *cipk8* mutant plants suggests that CIPK8 is involved in Arabidopsis salt tolerance by regulating Na^+^ export activity mediated by SOS1. Roles for CBL10 and SOS1 in promoting salt tolerance have been demonstrated in Arabidopsis (Quan *et al.*, 2007). These findings indicate that an additional arm of the SOS signaling pathway, CBL10–CIPK8–SOS1, functions to transport accumulated Na^+^ out of cells *in vivo* and is involved in promoting the salt tolerance of Arabidopsis.

When plants grow in saline soil, the roots directly interface with the saline environment, so Na^+^ exclusion by root cells is the initial defensive response utilized by plants to maintain cytoplasmic ion homeostasis. This response is critical for promoting plant salt tolerance. For example, in wheat roots, Na^+^ efflux rates should be high given that net uptake was very low compared with unidirectional influx (Davenport *et al.*, 2005). In both Arabidopsis and its halophytic relative *T. salsuginea*, 77–78% of the Na^+^ taken up by roots was subsequently recycled back out of the roots (Amtmann and Beilby, 2010). The Na^+^/H^+^ antiporter SOS1 is the only known Na^+^ efflux protein at the plasma membrane of plants thus far (Quintero *et al.*, 2011). The role of the SOS signaling pathways in regulating Na^+^ exclusion and plant salt tolerance is well established. In the plant roots, SOS3 can interact with SOS2, and the activated SOS3–SOS2 complex then stimulates Na^+^ extrusion through phosphorylation of SOS1; CBL10–SOS2 mainly regulates SOS1 activity in shoots, indicating that SOS2 is a key signaling node coordinating the response of roots and shoots to salt stress, such that both the SOS3–SOS2–SOS1 and CBL10–SOS2–SOS1 signaling pathways can simultaneously regulate Na^+^ exclusion in roots and shoots, playing a critical role in promoting plant salt tolerance. Given that the protein sequence, expression pattern, and subcellular distribution of CIPK8 are very similar to SOS2 (Figs 1, 2), it follows that the novel SOS pathway comprising CBL10, CIPK8, and SOS1 can also positively regulate plant salt tolerance via increased Na^+^ extrusion from cells. However, it was unexpected that both yeast two-hybrid and biomolecular complementation assays did not discover an interaction between SOS3 and CIPK8 (Fig. 6). SOS3 and CIPK8 could not increase the salt tolerance of yeast cells relative to a yeast strain expressing *SOS1* alone, indicating that SOS1 cannot be further activated by SOS3 and CIPK8. The CBL10-dependent and SOS3-independent regulatory pattern of CIPK8 on SOS1 activity indicates the regulatory effect of CIPK8 on Na^+^ efflux through SOS1 mainly occurs in the shoots. The *cipk8* loss of function mutant exhibited decreased growth under saline stress conditions, although the *sos2* mutant plants were more sensitive to NaCl treatment than the *cipk8* mutants. The fresh weight of *sos2* mutant plants was only 38% that of *cipk8* mutant plants when they were grown on MS plates supplied with 50 mM NaCl (see Supplementary Fig. S4). In saline soil containing 150 mM NaCl, the *sos2* mutants displayed a significantly more damaged phenotype than the *cipk8* mutants. The leaves of the *sos2* mutant turned dark brown, with some leaves becoming bleached as a result of salt treatment (Fig. 4). The above results suggest that SOS2 may more efficiently regulate the response of Arabidopsis plants to salinity stress than CIPK8. Thus, we can conclude that the major difference in salt tolerant functions between CIPK8 and SOS2 was due to lack of CIPK8 regulation of SOS1 in roots, since CIPK8-mediated activation of SOS1 relies on CBL10, not SOS3. These results suggest that SOS3 and CBL10 mediate distinct salt tolerance pathways in Arabidopsis (Yang *et al.*, 2019). SOS1 has been characterized as a transporter located in the root stellar tissues that regulates long-distance transport of Na^+^ from roots to shoots (Shi *et al.*, 2000; Olías *et al.*, 2009; El Mahi *et al.*, 2019). However, given that plant sensitivity to salinity is strongly associated with the accumulation of Na^+^ in the shoots (Coskun *et al*., 2013), the acropetal evacuation of Na^+^ mediated by SOS1 must be counteracted by exporting Na^+^ out of roots into the rhizosphere to some extent. It was recently reported in barley and wheat that SOS1-like amiloride-sensitive Na^+^ transporters at the xylem parenchyma interface might mediate the recirculation of Na^+^ by unloading Na^+^ from the root xylem (Zhu *et al.*, 2016, 2017). Here, ubiquitous expression of *CIPK8* driven by its own promoter was discovered in roots (Fig. 2), so it is possible that CIPK8 regulates Na^+^ extrusion mediated by SOS1 orthologues in other parts excluding the stellar tissue. Therefore, the reciprocally balanced Na^+^ extrusion activities between the root epidermis and stele could be controlled by two distinct pathways (CIPK8- and SOS3-dependent, respectively) in roots, and this regulation may be a key factor for promoting plant salt tolerance. However, this hypothesis regarding the relationship between CIPK8- and SOS3-dependent pathways requires further research.

## Supplementary data

Supplementary data are available at *JXB* online.

Fig. S1. The effect of NaCl treatment on the growth of Arabidopsis *sos1* and *sos2* seedlings.

Fig. S2. Phylogenetic tree analysis of Arabidopsis CIPK proteins.

Fig. S3. CIPK8 and SOS2 localized to the cytoplasm.

Fig. S4. The responses of *cipk8* and *sos2* to NaCl treatment.

Fig. S5. *CIPK8* expression partly rescues the salt sensitivity phenotype of *sos2cipk8.*

Fig. S6. The response of *sos2cipk8* and *sos1* to salt stress.

Fig. S7. The effect of NaCl treatment on the growth of *SOS1*- and *SOS1+CIPK8*- transgenic yeast cells.

Fig. S8. Salt tolerance tests of the yeast mutant strain AXT3K expressing *SOS1* with or without *CIPK8* and combinations of *CBLs*.

Table S1. Primers used in this study.

erz549_suppl_Supplementary_Material

## Data deposition

Sequence data from this article can be found at GenBank under the following accession numbers: *ACTIN*, AT3G18780; *SOS1*, AT2G01980; *SOS2*, AT5G35410; *CBL1*, AT4G17615; *CBL2*, AT5G55990; *CBL3*, AT4G26570; *CBL4*, AT5G24270; *CBL5*, AT4G01420; *CBL6*, AT4G16350; *CBL7*, AT4G26560; *CBL8*, AT1G64480; *CBL9*, AT5G47100; *CBL10*, AT4G33000.
