# Supplementary material for: The protein kinase complex CBL10–CIPK8–SOS1 functions in Arabidopsis to regulate salt tolerance
Source: J Exp Bot. 2019 Dec 10;71(6):1801–14. doi: 10.1093/jxb/erz549 (PMC7242078; doi:10.1093/jxb/erz549)
Supplement: erz549_suppl_Supplementary_Material [file erz549_suppl_supplementary_material.pdf]

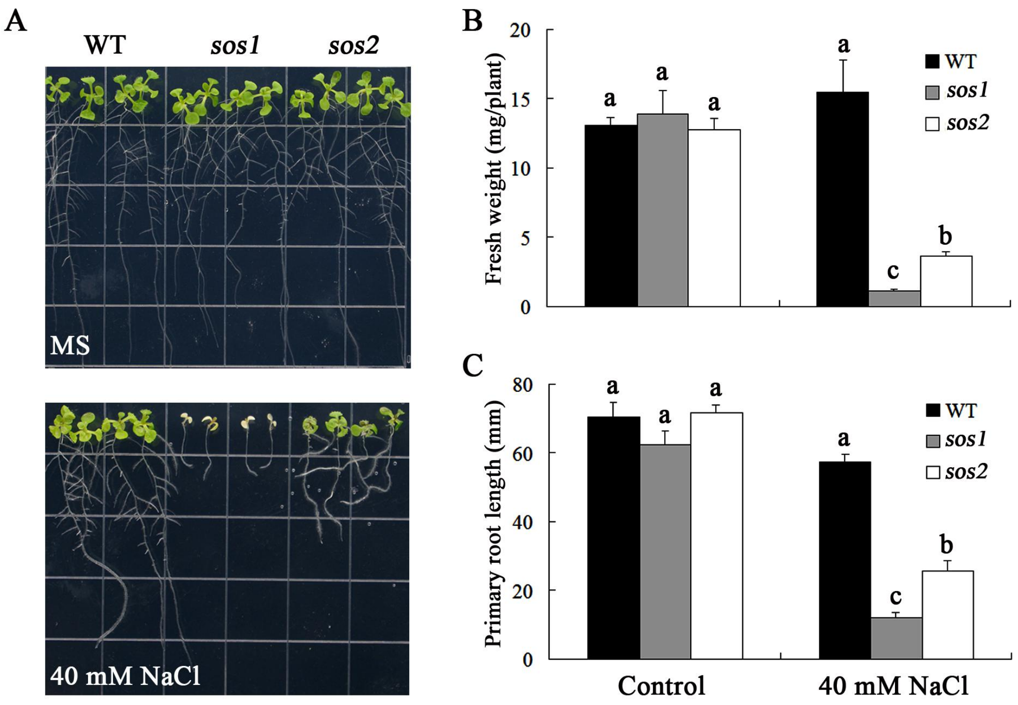

Fig. S1. The effect of NaCl treatment on the growth of *Arabidopsis sos1* and *sos2* seedlings. (A) Five-day-old wild-type, *sos1* and *sos2* seedlings were transferred onto MS plates containing 0 and 40 mM NaCl, respectively. The pictures were taken after 7 d treatment. Fresh weight (B) and primary root length (C) were measured at day 7 after NaCl treatment. Values are mean ± SE (n=12) and different letters above the columns indicate significant differences at $p < 0.05$ level among the different data.

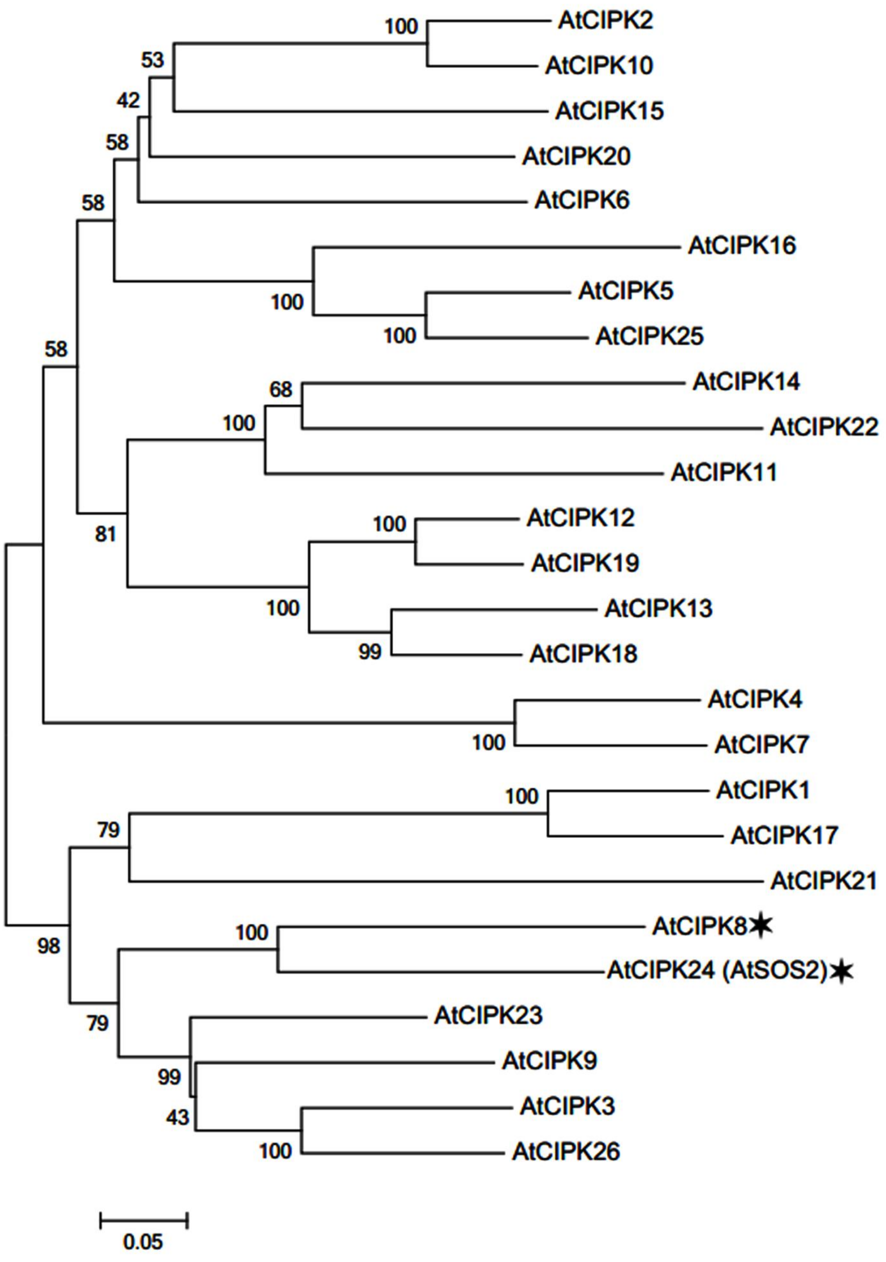

Fig. S2. Phylogenetic tree analysis of *Arabidopsis thaliana* CIPK proteins.

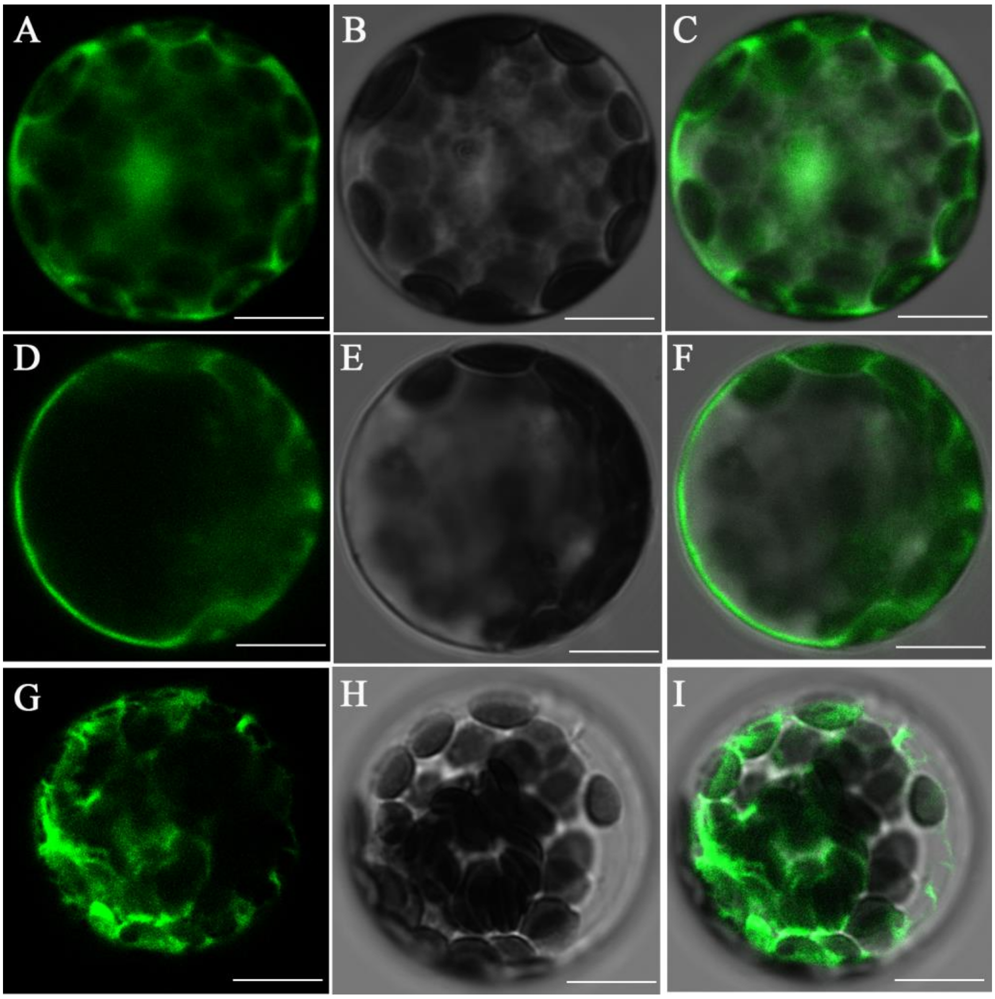

Fig. S3. CIPK8 and SOS2 localize to the cytoplasm. (A) to (C) Fluorescent signal of *Arabidopsis* mesophyll protoplast transformed with pCAMBIA1300-*GFP*. (D) to (F) Fluorescent signal of *Arabidopsis* mesophyll protoplast transformed with pCAMBIA1300-*CIPK8-GFP*. (G) to (I) Fluorescent signal of *Arabidopsis* mesophyll protoplast transformed with pCAMBIA1300-*SOS2-GFP*. Bars = 10 μm.

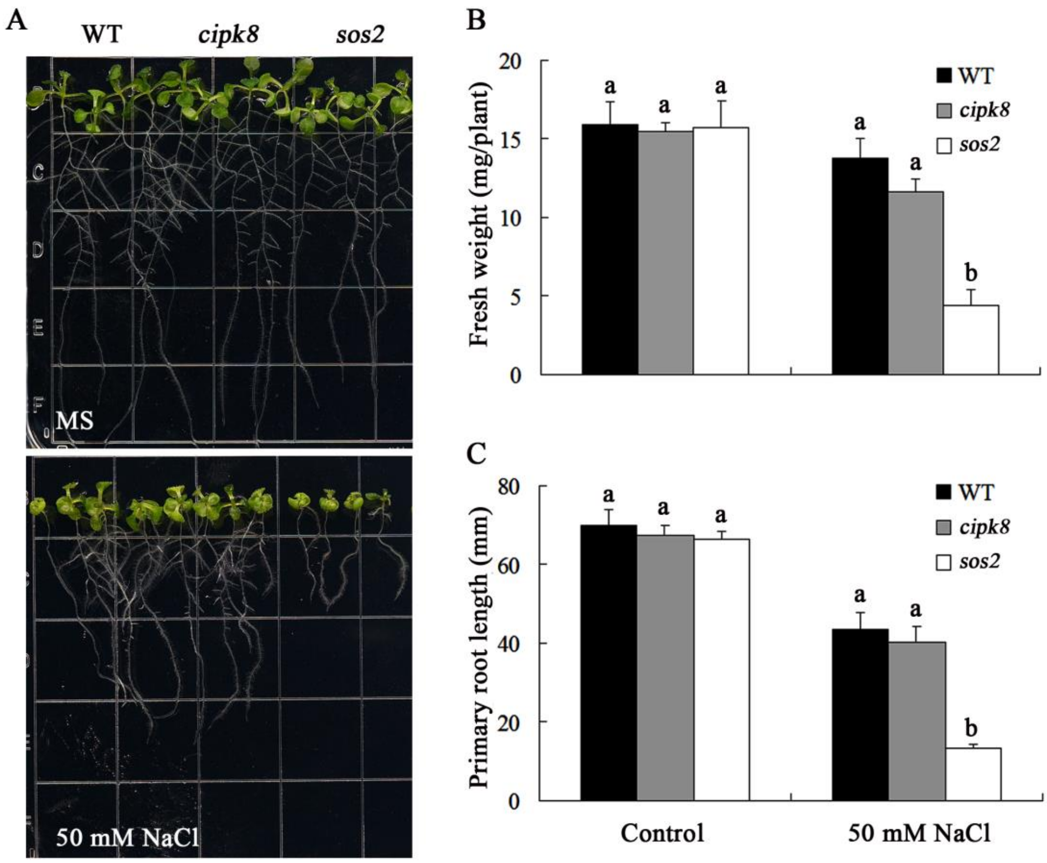

Fig. S4. The responses of *cipk8* and *sos2* to NaCl treatment. (A) WT, *cipk8* and *sos2* seedlings were photographed after 7 days of NaCl treatment. Fresh weights (B) and primary root lengths (C) of WT, *cipk8* and *sos2* were measured at day 7 after NaCl treatment. Values are mean ± SE (n=12) and different letters above the columns indicate significant differences at $p < 0.05$ level among the different data.

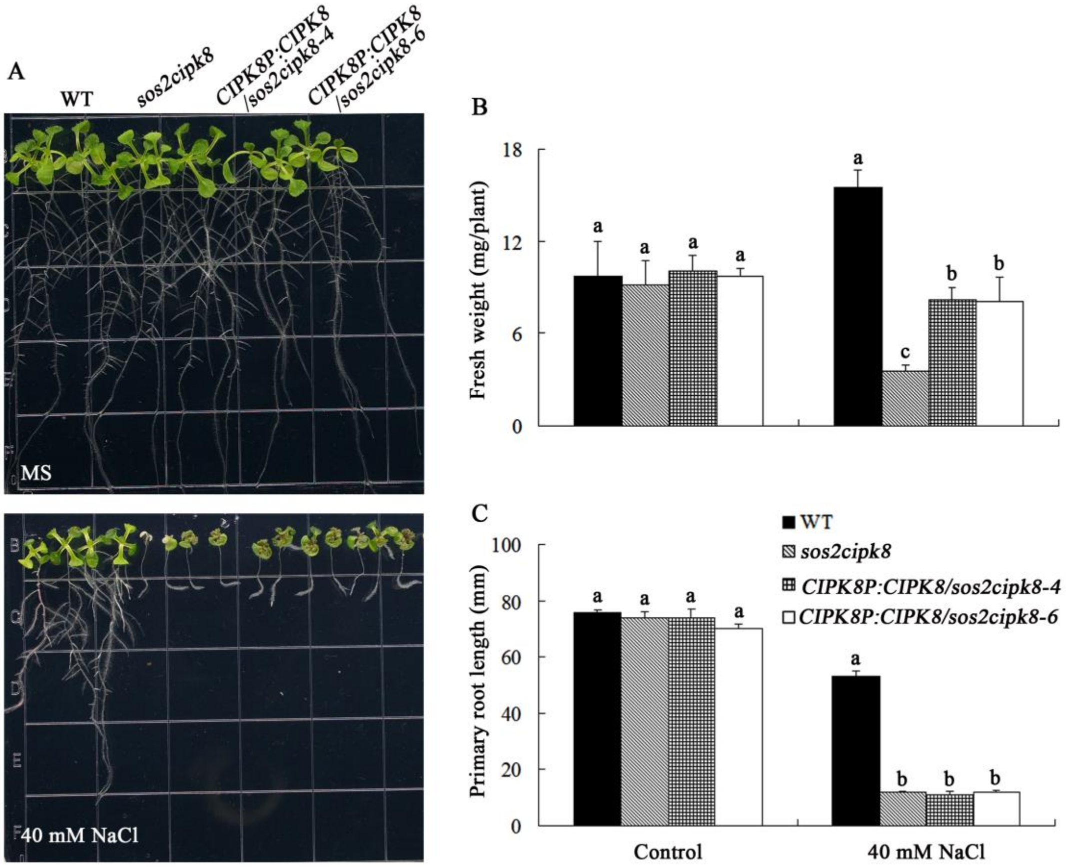

Fig. S5. *CIPK8* expression partly rescues the salt sensitivity phenotype of *sos2cipk8.* A 5502 bp of DNA fragment containing *CIPK8* promoter and *CIPK8* gene (*CIPK8P*:*CIPK8*) was introduced into *sos2cipk8* double mutant plants through mediation of vector pCAMBIA1300. Eight of *CIPK8*-transgenic homozygote lines (*CIPK8P*:*CIPK8*/*sos2cipk8*) were screened from T3 generations. Two transgenic lines, *CIPK8P:CIPK8*/*sos2cipk8*-4 and *CIPK8P:CIPK8*/*sos2cipk8*-6, were used for the followed experiments. The pictures were taken after seedlings of WT, *sos2cipk8* and *CIPK8P:CIPK8*/ *sos2cipk8* plants grew under salt stress for 7 days (A), and then the fresh weights (B) and primary root lengths (C) were determined. Data represent the mean ± SE of twelve replicates and different letters above the columns indicate significant differences at $p < 0.05$ level among the different data.

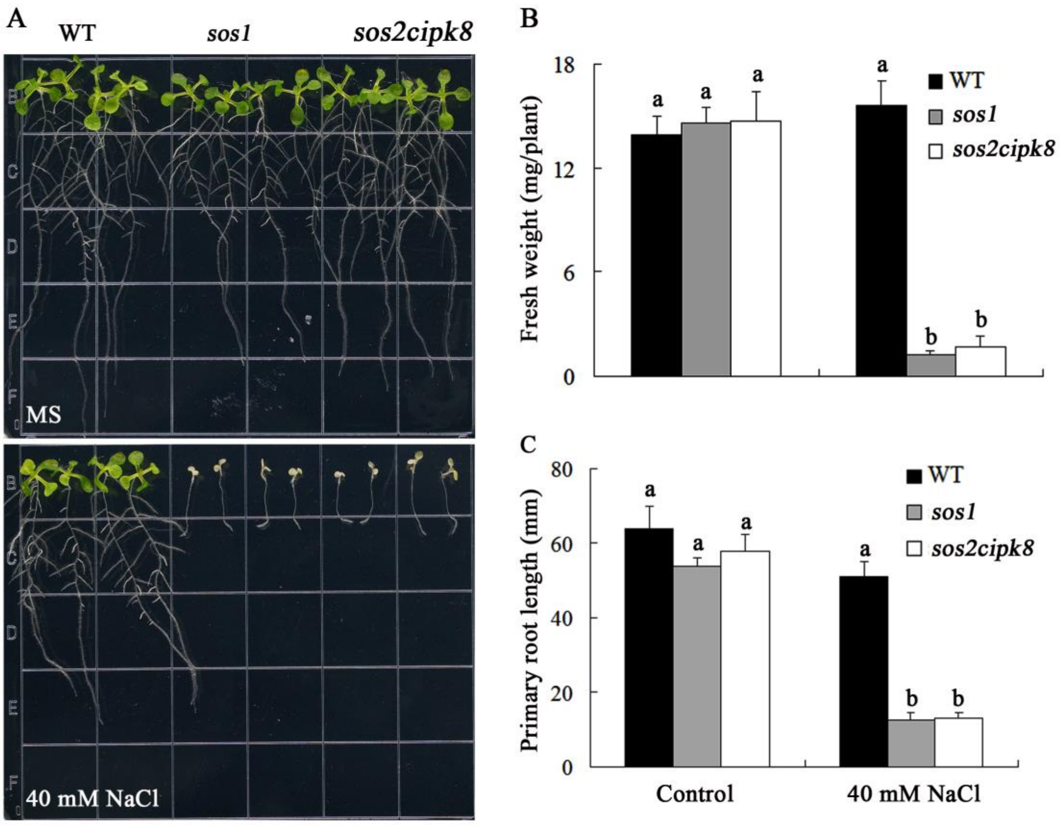

Fig. S6. The response of *sos2cipk8* and *sos1* to salt stress. (A) WT, *sos1* and *sos2cipk8* seedlings were photographed after 7 days of NaCl treatment. Fresh weights (B) and primary root lengths (C) of WT, *sos1* and *sos2cipk8* after 7 days of NaCl treatment. Values are mean ± SE (n=12) and different letters above the columns indicate significant differences at $p < 0.05$ level among the different data.

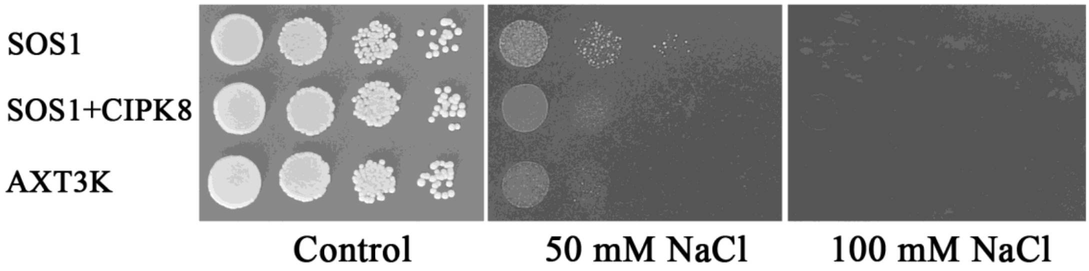

Fig. S7. The effect of NaCl treatment on the growth of *SOS1*- and *SOS1+CIPK8*-transgenic yeast cells.

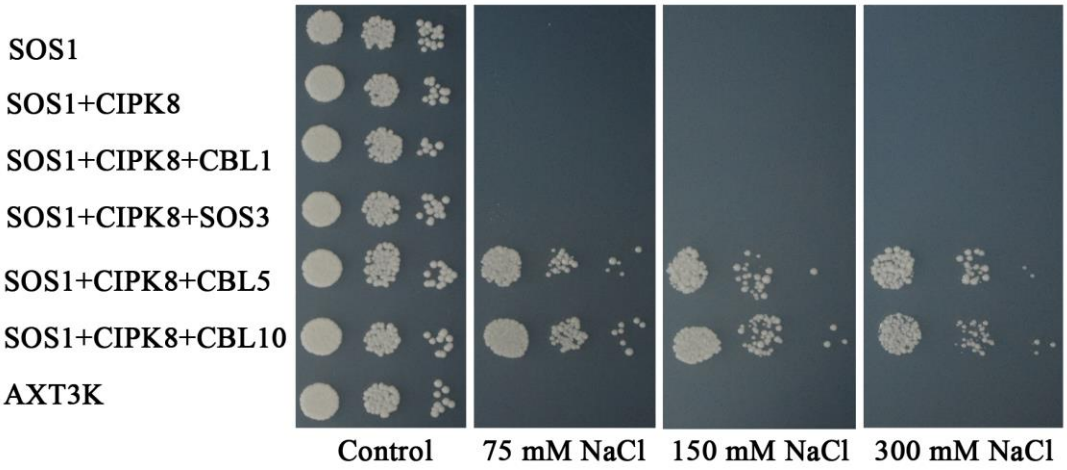

Fig. S8. Salt tolerance tests of the yeast mutant strain AXT3K expressing *SOS1* with or without *CIPK8* and combinations of *CBLs*. *SOS1* was cloned into the plasmid p416 (p416-*SOS1*); *CIPK8* and related *CBLs* were cloned into p414 (p414-*CIPK8*-*CBLs*) as described in the section "Materials and methods". p416-*SOS1* alone or p416-*SOS1* and p414-*CIPK8*-*CBLs* together were transformed into AXT3K, respectively. Transgenic and untransformed yeast cells were spotted on AP plates with or without NaCl as described in the section "Materials and methods", and cultured at 28°C for 3-5 days.

Supplemental Table S1. Primers used in this study.

Purpose	Primer	Primer sequence
Identification of cipk8 mutant plants	LP	GAATCAAGAGGCAACAACAGC
	RP	ATCGTGGATCGTAGCACAATC
	LBb1.3	ATTTTGCCGATTTCGGAAC
RT-PCR primers of SOS2	SOS2-RT-F	CGGGATCCATGACAAAGAAAATGAGAAG
	SOS2-RT-R	GTTCCACATGTGGTACGCAGAAGTTC
RT-PCR primers of CIPK8	CIPK8-RT-F	TTGGCCTCAGTGCATTACCT
	CIPK8-RT-R	TAGTGGACCTGTGTCTCTTG
RT-PCR primers of β-Actin	Actin-RT-F	GAGGTTACATGTTCACCACAACA
	Actin-RT-R	CCTGATATCCACATCACACTTCA
qRT-PCR primers of CIPK8	CIPK8-qRT-F	TTGGCCTCAGTGCATTACCT
	CIPK8-qRT-R	CGGCACCATTGTAACCCTTG
qRT-PCR primers of β-Actin	Actin-qRT-F	TATGAATTACCCGATGGGCAAG
	Actin-qRT-R	TGGAACAAGACTTCTGGGCAT
CIPK8-GFP fusion	CIPK8-GFP-F	ACGCGTCGACATGGTGGTAAGGAAGGTG
	CIPK8-GFP-R	GGACTAGTACGTCTTTTACTC
SOS2-GFP fusion	SOS2-GFP-F	ACGCGTCGACATGACAAAGAAAATGAGAAGAGTG
	SOS2-GFP-R	GGACTAGTAAACGTGATTGTTCTGAGAATCTC
CIPK8 localization	CIPK8-221-F	GGGGACAAGTTTGTACAAAAAAGCAGGCTTAATGGTGGTAAGGAAGGTGGGC
	CIPK8-221-R	GGGGACCACTTTGTACAAGAAAGCTGGGTCTCAACGTCTTTTACTCTTGG
SOS2 localization	SOS2-221-F	GGGGACAAGTTTGTACAAAAAAGCAGGCTTAATGACAAAGAAAATGAGAAG
	SOS2-221-R	GGGGACCACTTTGTACAAGAAAGCTGGGTCTCAAAACGTGATTGTTCTGAG

CIPK8 promoter	CIPK8P-F	GGGGTACCAAGAGGAGGAGGAGGAG
	CIPK8P-R	CATGCCATGGATTCTCTTCTGCTTCTAC
CIPK8P:CIPK8 for complementation	CIPK8P-CIPK8-F	GGGGTACCGTTCTCCCTGAGGCACATT
	CIPK8P-CIPK8-R	ACGCGTCGACTCAACGTCTTTTACTCTTGG
For yeast two-hybrid system	CIPK8-BD-F	CGGAATTCATGGTGGTAAGGAAGGTG
	CIPK8-BD-R	ACGCGTCGACGTTCAACGTCTTTTACTC
	CBL1-AD-F	CGGAATTCATGGGCTGCTTCCACTCAAAG
	CBL1-AD-R	CGCGGATCCTCATGTGGCAATCTCATCG
	CBL2-AD-F	CGGAATTCATGTCGCAGTGCGTTGAC
	CBL2-AD-R	CGCGGATCCTCAGGTATCTTCAACCTGAG
	CBL3-AD-F	CGGAATTCATGTCGCAGTGCATAGACG
	CBL3-AD-R	CGCGGATCCTCAGGTATCTTCCACCTGC
	CBL4-AD-F	CGGAATTCATGGGCTGCTCTGTATCGAAG
	CBL4-AD-R	CCGCTCGAGTTAGGAAGATACGTTTTGC
	CBL5-AD-F	CGGAATTCATGGGATGTGTTTGCAGCA
	CBL5-AD-R	CGCGGATCCTTACCGGAGAAAGGTTGGG
	CBL6-AD-F	CGGAATTCATGATGATGCAATGTTTAGATGG
	CBL6-AD-R	CGCGGATCCTCATCCATCCAGCTCACTAGGAGT
	CBL7-AD-F	CGGAATTCATGGATTCAACAAGAAATTC
	CBL7-AD-R	CGCGGATCCTCAGGTATCTTCCACTTGC
	CBL8-AD-F	CGGAATTCATGTTGGCATTCGTGAAATG
	CBL8-AD-R	TCCCCCGGGCTAGTCTTCAACTTCAGAGTCGAGT
	CBL9-AD-F	CGGAATTCATGGGTTGTTTCCATTCCACGG
	CBL9-AD-R	CGCGGATCCTCACGTCGCAATCTCGTCC
	CBL10-AD-F	CGGGATCCGTATGGAACAAGTTTCCTCTAG

	CBL10-AD-R	CCGCTCGAGCGTCAGTCTTCAACCTCAGTGT
Split-LUC assay	CIPK8-cLUC-F	GGGGTACCATGGTGGTAAGGAAGGTG
	CIPK8-cLUC-R	ACGCGTCGACTCAACGTCTTTTACTCT
	SOS2-cLUC-F	GGGGTACCATGACAAAGAAAATGAGAAGAGT
	SOS2-cLUC-R	ACGCGTCGACTCAAAACGTGATTGTTC
	SOS3-nLUC-F	TCGGTACCCGGGATCCaATGGGCTGCTCTGTATCGAAG
	SOS3-nLUC-R	ACGAGATCTGGTCGACGGAAGATACGTTTTGC
	CBL10-nLUC-F	TCGGTACCCGGGATCCaATGACAACTGGCCGA
	CBL10-nLUC-R	ACGAGATCTGGTCGACGTCTTCAACCTCAGTG
Yeast complementation test	SOS1-p416-F	GCTCTAGAATGACGACTGTAATCGACGCG
	SOS1-p416-R	TCCCCCGGGTCACTGATCAGAGCTTGAGCTAC
	CIPK8-p414-F	CGGGATCCATGGTGGTAAGGAAGGTG
	CIPK8-p414-R	ACGCGTCGACGTTCAACGTCTTTTACTC
	SOS2-p414-F	CGGGATCCATGACAAAGAAAATGAGAAGAGT
	SOS2-p414-R	CGGAATTCTCAAAACGTGATTGTTC
	CIPK8-F	ACTAGTATGGTGGTAAGGAAGGTGGGC
	CIPK8-CYC1-R	ATAACTAATTACATGATCAACGTCTTTTACTCTTG
	CYC1-CIPK8-F	CAAGAGTAAAAGACGTTGATCATGTAATTAGTTAT
	CYC1-R	GCGGATCCGGCCGCAAATTAAAGCCTTC
	SOS2-F	GACTAGTATGACAAAGAAAATGAGAAG
	SOS2-CYC1-R	ATAACTAATTACATGATCAAAACGTGATTGTTC
	CYC1-SOS2-F	GAACAATCACGTTTTGATCATGTAATTAGTTAT
	GPD-F	GCGGATCCAGTTTATCATTATCAATAC
	GPD-CBL1-F	CTTTGAGTGGAAGCAGCCCATATCCGTCGAAACTAAG
	CBL1-GPD-R	CTTAGTTTCGACGGATATGGGCTGCTTCCACTCAAAG

	CBL1-R	ATAGTCGACTCATGTGGCAATCTCATCG
	GPD-CBL4-F	CTTCGATACAGAGCAGCCCATATCCGTCGAAACTAAG
	CBL4-GPD-R	CTTAGTTTCGACGGATATGGGCTGCTCTGTATCGAAG
	CBL4-R	ATAGTCGACTTAGGAAGATACGTTTTGC
	GPD-CBL5-F	TGCTGCAAACACATCCCATATCCGTCGAAACTAAG
	CBL5-GPD-R	CTTAGTTTCGACGGATATGGGATGTGTTTGCAGCA
	CBL5-R	ATAGTCGACTTACCGGAGAAAGGTTGGG
	GPD-CBL10-R	GGTCGGCCAGTTGTCATATCCGTCGAAACTAAG
	CBL10-GPD-F	CTTAGTTTCGACGGATATGACAACTGGCCGACC
	CBL10-R	ATAGTCGACTCAGTCTTCAACCTCAGTG
Site-mutations of SOS1	SOS1-p416-F	GCTCTAGAATGACGACTGTAATCGACGCG
	1136A-p416-R	CGGGGTACCTCATAGATCGTTCCTGAAAACGATTTTACTCGGagcgTCGATTCTCACAACGATT
	1138A-p416-R	CGGGGTACCTCATAGATCGTTCCTGAAAACGATTTTcgcCGGcGAATCGATTCTCACAACGATT
	1136A1138A-p416-R	CGGGGTACCTCATAGATCGTTCCTGAAAACGATTTTcgcCGGcgcgTCGATTCTCACAACGATT
